# Decomposing dynamical subprocesses for compositional generalization

**DOI:** 10.1073/pnas.2408134121

**Published:** 2024-11-08

**Authors:** Lennart Luettgau, Tore Erdmann, Sebastijan Veselic, Kimberly L. Stachenfeld, Zeb Kurth-Nelson, Rani Moran, Raymond J. Dolan

**Affiliations:** ^a^Imaging Neuroscience, Max Planck UCL Centre for Computational Psychiatry and Ageing Research, University College London, London WC1B 5EH, United Kingdom; ^b^Imaging Neuroscience, Wellcome Centre for Human Neuroimaging, University College London, London WC1N 3AR, United Kingdom; ^c^Division of Psychiatry, Faculty of Brain Sciences, University College London, London W1T 7NF, United Kingdom; ^d^Clinical and Movement Neurosciences, Department of Motor Neuroscience, University College London, London WC1N 3BG, United Kingdom; ^e^Google DeepMind, London N1 C4AG, United Kingdom; ^f^Science and Engineering Department, School of Biological and Behavioural Sciences, Queen Mary University of London, London E1 4DQ, United Kingdom

**Keywords:** structure learning, memory, generalization, abstraction, compositionality

## Abstract

A striking feature of human cognition is an exceptional ability to rapidly adapt to novel situations. It is proposed this relies on abstracting and generalizing past experiences. While previous research has explored how humans detect and generalize single sequential processes, we have a limited understanding of how humans adapt to more naturalistic scenarios, for example, complex, multisubprocess environments. Here, we propose a candidate computational mechanism that posits compositional generalization of knowledge about subprocess dynamics. In two samples (*N* = 238 and *N* = 137), we combined a novel sequence learning task and computational modeling to ask whether humans extract and generalize subprocesses compositionally to solve new problems. In prior learning, participants experienced sequences of compound images formed from two graphs’ product spaces (group 1: G1 and G2, group 2: G3 and G4). In transfer learning, both groups encountered compound images from the product of G1 and G3, composed entirely of new images. We show that subprocess knowledge transferred between task phases, such that in a new task environment each group had enhanced accuracy in predicting subprocess dynamics they had experienced during prior learning. Computational models utilizing predictive representations, based solely on the temporal contiguity of experienced task states, without an ability to transfer knowledge, failed to explain these data. Instead, behavior was consistent with a predictive representation model that maps task states between prior and transfer learning. These results help advance a mechanistic understanding of how humans discover and abstract subprocesses composing their experiences and compositionally reuse prior knowledge as a scaffolding for new experiences.

## Significance

Humans possess a remarkable ability to adapt rapidly and flexibly to novel situations, a key aspect of cognition. While past studies detail how we learn from single processes, we have limited understanding of how we navigate complex real-world environments, where multiple simultaneous processes interact. Using a sequence learning task, we show that humans segregate holistic experiences into component parts and deploy this knowledge in similar tasks by recombining these component parts. We detail a predictive model that explains how subjects generalize this learned information to new scenarios. Our findings offer insight into psychological mechanisms behind human cognitive adaptability, enhancing an understanding of how we adapt effectively to novel environments.

Human cognition is characterized by an exceptional ability to rapidly adapt to novel situations, and it is suggested this is driven by robust generalization abilities, where these allow transfer of prior knowledge to new situations (1–3). More broadly, it has been proposed that generalization contributes to human performance that exceeds many AI systems (3–9).

Much of our experiences of the world reveal themselves as sequential and temporally unfolding events. An extensive literature, in psychology and cognitive science, on generalization abilities has focused on serially organized behaviors (10–12). Using ordinal list learning tasks (e.g., simultaneous presentation of multiple items to select from in correct sequential order) and derived lists paradigms (compiling novel lists from known list items that retain the trained ordinal item positions) it has been shown, across species and developmental stages, that learners do not simply encode associations between adjacent list items, but also learn abstract positional information, knowledge that is generalizable to novel learning scenarios (10–16). This flexible use of abstract knowledge in novel contexts is further enhanced if the ordinal positions of items correspond to an increasing number of elements per item [e.g., the number of shapes contained in a stimulus (10–12), increasing item size (10–12, 15), or increasing complexity of stimuli (17)].

In artificial grammar learning tasks, it has been shown that humans extract hidden rules (although see ref. 18 for comprehensive review of potential cognitive mechanisms) of stimulus sequences generated by finite state machines (18). They also readily learn different types of artificial grammars, irrespective of stimulus modality (visual, auditory, tactile) (18–21), extracting both low-level (e.g., relative frequency of a stimulus) and higher-order statistics (e.g., joint probability of stimuli) from visual images (22). Additionally, within the same modality humans generalize knowledge of artificial grammars selectively to new stimuli that share the same statistical regularities, but not to stimuli that do not adhere to these same rules (23). This has led to a suggestion that artificial grammar learning is supported by a specific, modality-constrained, representation (24).

A related line of research on sequential learning has examined how subjects extract and (neurally) represent statistical regularities when experiencing a sequentially unfolding process (25–29) or piecemeal presentation of a graph structure (30). Neural evidence suggests the hippocampal-entorhinal system represents spatial, temporal (31), and semantic (32) aspects of on-going experience, indicating graph structured representations are a fundamental organizing principle of memory. Of note, previous studies exclusively focused on sequential learning and behavior driven by a single dynamical process (but see ref. 24, which investigated mixed, temporally separated, stimulus sequences produced by two artificial grammars).

However, our everyday experiences of the world are invariably the product of complex environmental dynamics, composed and generated from multiple, simultaneously evolving subprocesses—though for the most part even the most complex novel experiences share structural similarities with previous experiences. These properties of the world may allow reuse of discovered abstracted knowledge of relational dynamics in new contexts. Given the ubiquity of such complex dynamics it is surprising how little is known about the cognitive mechanisms that enable parsing of subprocess dynamics of experience and the generalization of such knowledge to novel situations.

A plausible mechanism enabling this behavior is compositional generalization of prior knowledge, involving breaking down complex tasks into small “building blocks” and recombining these to solve new problems. Here, we acknowledge different usages of the term compositional across different subfields of cognitive (neuro)science, for instance recursive operations over hierarchies [e.g., in language, repeatedly applying parsing rules to different levels of a sentence structure, starting at the highest level (the entire sentence) and moving down to the lowest levels (individual words), which systematically breaks down a sentence into its component parts] (33, 34), in hierarchical function composition (function A generates output A which is used as input to function B, generating output B), or in rich forms of language semantic composition to create complex concepts (35). Here, we use a specific understanding of the term, inspired by definitions of compositionality used in recent cognitive-computational neuroscience studies (e.g., refs. 36–38). This usage of composition involves its simplest, most literal, and least technical sense, to capture the process of creating a new entity from combining two or more things. In the specific context of our study, composition refers to the joint dynamics of subprocesses that produce sequences of stimulus combinations.

However, thus far, compositional generalization has been investigated primarily in static, nondynamical, settings (3, 5, 7, 36) leaving open a question of whether and how compositional generalization is realized in dynamical settings. These knowledge gaps are highly relevant for a range of naturalistic tasks humans solve on a daily basis, where multiple, simultaneously acting, subprocesses need to be extracted from complex environmental dynamics.

Consider, by way of example, a scenario where you are watching a TV chef cooking a meal where you want to learn how to cook a new dish for dinner while, at the same time, you are also listening to the radio. The events that unfold are likely to include observing a sequence of increasing the applied heat, the introduction of novel ingredients, followed by a decrease in the heat coupled with periodic stirring—and all of this happening while you are simultaneously listening to music, the weather forecast, and the traffic news in their turn. Both processes—learning how to prepare dinner from the TV chef and listening to the radio jointly contribute to on-going experience. In principle, we could learn the entire complicated temporal trajectory of the evening’s progression as a holistic entity—the joint experience of observing the TV chef’s cooking routine and hearing changing radio programs—or, alternatively, learn separately the dynamics of preparing the dish and the structure of the radio program. Only the second of these enables an agent to generalize component elements to new experiences that share some, but not all, subcomponents. For example, the latter would support reasoning that preparing a different meal at a friend’s place is likely to first involve increasing the applied heat, followed by an introduction of novel ingredients, all while chatting to the friend—or equally allow us to reliably predict that driving and listening to the radio the weather forecast will be followed next by traffic news.

**Fig. 1.**
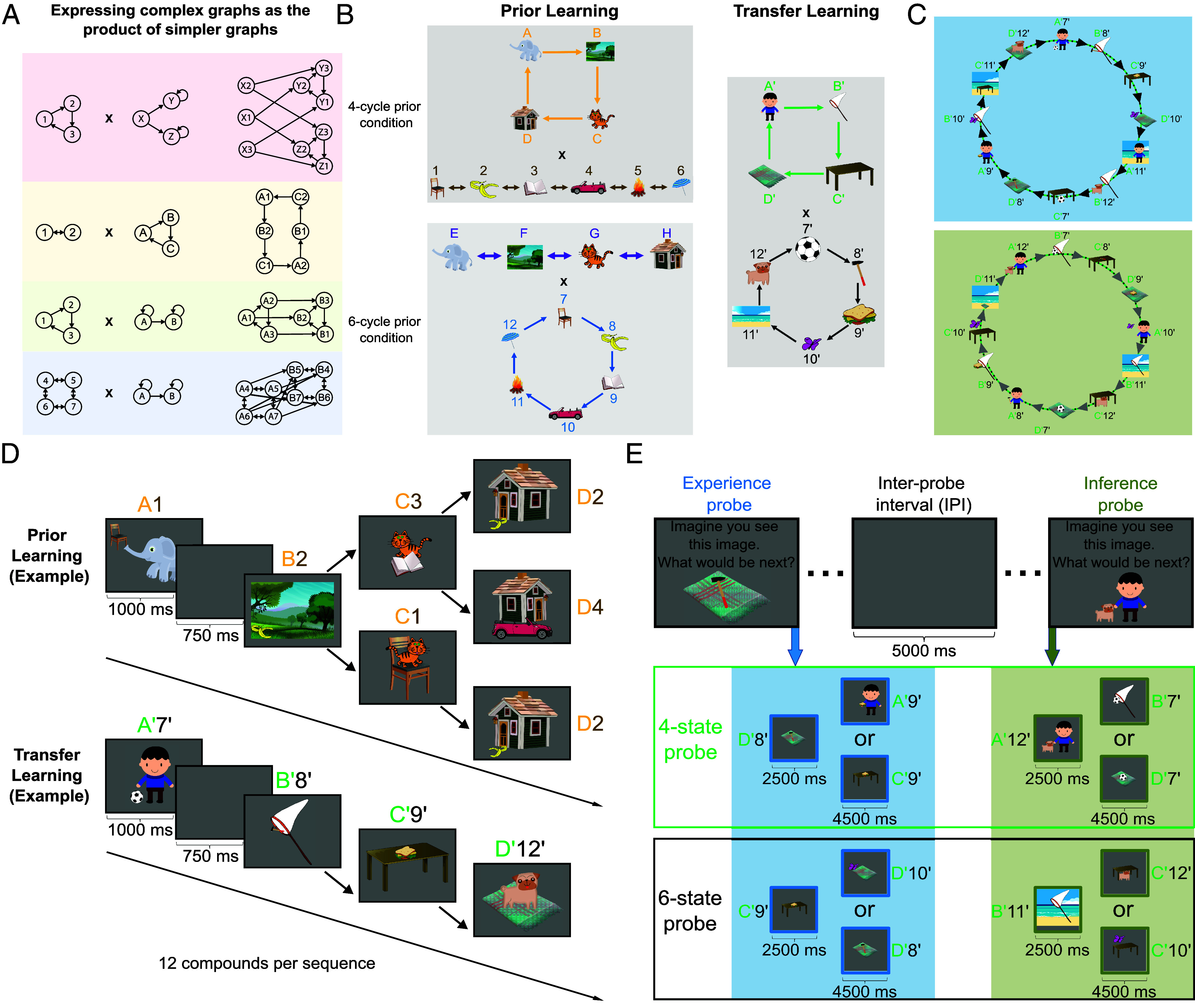
Experimental design and behavioral task. (*A*) Exemplar product graphs (Cartesian product) drawn from a large space of potential graph products (*Right*), that are composed from two simpler graph factors (*Left*). The graph product of two or more simple graph factors creates complex product state spaces that can be factored (and simplified) into the constituting factors. (*B*) Set of graph factors selected for prior learning (*Left*) and transfer learning (*Right*). (*C*) For the sequence learning task (prior and transfer learning), graph factors were composed to compound graphs (product graphs) by combining their images. For illustrative purposes, we only display the compound graph for transfer learning, produced from the 4-cycle and 6-cycle graphs depicted in panel *B* (*Right*). The compound graphs in both task phases (prior and transfer learning) comprised two disconnected subgraphs of 12 states. During sequence presentations in transfer learning, participants only observed compound images from one of these subgraphs (panel *C Top*) and consequently only saw 12 transitions between compound images, out of a full set of 24 possible transitions. The remaining 12 compound images provided for a set of held-out compound stimuli (panel *C*, *Bottom*, “inferred subgraph”) that allowed us to test knowledge of unobserved transitions. Thus, participants *never* observed transitions between held-out compound stimuli from the inferred subgraph. The same principles applied to prior learning as well. (*D*) Exemplar sequences produced by the product graphs. During both prior learning and transfer learning, participants observed sequences of compound images. Each compound was a meaningful holistic scene, for example, a boy with a ball or a dog on a blanket. Each sequence comprised 12 compound images. Following each sequence presentation (12 compound stimuli), participants’ understanding of temporal dynamics along these sequences was probed in two ways, as described in panel *E*. In a between-subjects design two groups of participants (*N* = 238 total) first underwent a prior learning phase (*Left* column; 48 sequences of 12 compound images). Participants in a 4-state cycle prior condition experienced sequences generated from combining a 4-state cyclic graph factor and a 6-state path-graph factor (panel *B*, *Top* row). Participants in a 6-state cycle prior condition saw compound image sequences produced by combining a 4-state path-graph factor and a 6-state cyclic graph factor (panel *B*, *Bottom* row). Traversals on the path-graphs were probabilistic. At each nonboundary state, participants could transition, with equal probability, either to the state on the *Left* or *Right*. The specific order of images contributed to by the 4-cycle (or 6-cycle) remained constant across the 48 sequence presentations in prior learning. Only the order of images derived from the path-graphs changed across the 48 sequences. The prior learning phase was followed by a transfer learning phase, identical for both conditions (*Right* column; 36 repetitions of the same sequence consisting of 12 compound images) and featuring 12 entirely new compound images composed from 10 new individual images, none of which appeared in the prior learning phase. During this transfer learning phase, the compound image sequence was generated by combining two cyclic graphs (4 and 6 states). For both prior learning conditions, only one of the two prior learning abstract graph factors was consistent across prior and transfer learning (e.g., the 4-state cycle in the 4-state cycle prior condition). Importantly, prior and transfer learning phase sequences presented participants with entirely different compound images, and the sequential presentation of these compound images followed different underlying dynamics (i.e., they were produced by the combination of distinct graph factors). (*E*) During both prior and transfer learning, following each presented sequence, participants were probed to predict upcoming states that followed one of the 12 experienced compound images (experience probes, *Left* column). Additionally, an inference probe (*Right* column) tested participants’ ability to infer and predict transitions between the 12 held-out compounds (panel *C*, *Bottom*). In each probe question, participants first saw a randomly selected compound image from the experienced sequence (or a held-out compound image, respectively) and were then asked: “Imagine you see this image. What would be the next image?” Next, two compound images—the correct next compound image and a lure compound image—were presented as choice options. The lure compound image was matched with the correct option on one of the two individual images composing the compound image (e.g., D’8’ -> A’9’ or C’9’). This allowed us to test knowledge of both graph factors separately (*Top* row: 4-state factor; *Bottom* row: 6-state factor) underlying the observed sequence (and the held-out compound images) separately.

While we take for granted that our experience of the world arises from the interplay of multiple distinct, and simultaneously unfolding, dynamical subprocesses, there is little empirical evidence showing that humans decompose their experience into dynamical subprocesses, let alone recompose these for new learning. One way of approaching this problem is by considering product graphs, where the joint dynamics (Cartesian product) of two or more graph factors create complex product state space dynamics that can be factored (and simplified) into constitutive factors. Graph factorization thus provides a compelling and flexible test bed for probing compositional generalization of multiple (independent) and simultaneously unfolding subprocesses. Examples for such graph products from the vast space of possible combinations are provided in Fig. 1*A*.

Cognitive-computational models for structural inference, such as the Tolman-Eichenbaum Machine (39) and Clone-Structured Cognitive Graphs (40), have detailed likely cognitive (and neural) processes that enable building and exploitation of a cognitive world map in the service of efficient generalization of knowledge (2). Here, rapid inference enables factoring structure from sensory details, for example by representing separately knowledge of abstract generative processes and specific encountered stimuli. However, these models primarily speak to the special case where a single structural form underlies experience. For example, Mark et al. (41) examined the transfer of global and entire graph structures and found that humans generalize learned graph structures to new situations. While attributing experience to a single structural process can generalize to structurally identical novel contexts it cannot easily do so in more realistic learning scenarios where there is only a partial overlap between current environmental processes and those abstracted from past situations.

A class of computational models that bear on this question relates to learning predictive representations of environment dynamics from temporal contiguity of experienced task states [successor feature models (42–44)]. Notably, these models support a representation of complex environmental dynamics generated from multiple simultaneous subprocesses. However, such models struggle with transferring knowledge to novel contexts (41, 43), because learned representations are highly specific to experiential contexts. Thus, encountering new features/states (in a novel situation) necessitates learning entirely new representations from scratch. Here, we present a predictive representation model architecture that overcomes this limitation and detail a relatively simple computational mechanism that can allow transfer of learned representations across contexts. This model has some resemblance to cognitive accounts of analogical reasoning (structure mapping theory) where it is proposed past experiences serve as an analogy to solve highly distinct, but structurally similar, future problems (45, 46). Our model is capable of reusing prior knowledge by learning a mapping between previously and currently encountered temporal dynamics, with prior knowledge serving as a “scaffolding” of incoming new information.

Here, we provide support for a hypothesis that humans extract multiple subprocesses from complex dynamical, sequentially unfolding, experiences and reuse this knowledge in a compositional manner to facilitate learning in novel situations. We show that experiencing temporal dynamics in an initial learning phase helps predict temporal relationships in a subsequent transfer learning phase, selectively for (dynamical) components that were experienced consistently across the two learning phases. Predictive representation models, based solely on the temporal contiguity of experienced task states specific to a given context, failed to capture the behavioral data features observed in human transfer learning performance. Instead, behavior was consistent with predictions of a transfer learning predictive representation model. We suggest this form of knowledge transfer confers computational and cognitive advantages, including facilitating discovery of structurally identical subprocesses to enable efficient structuring of learning and the compositional reuse of prior experience.

## Results

We acquired two independent online samples of healthy human volunteers (*N* = 238 in a main sample and *N* = 137 in a replication sample). Since both samples yielded highly similar results, we focus our report on the results of the main sample and refer readers to *SI Appendix*, Fig. S1 for description of consistent results observed in the replication sample.

In the experimental task, participants viewed sequences of compound images, each compound depicting a meaningful holistic scene comprising two parts, for example, a boy with a ball or a dog on a blanket (Fig. 1*D*). These sequences were derived from walks on a graph (Fig. 1 *C*, *Top*). Crucially, the underlying graph had a structural property, not communicated to participants, such that it could be factored into two simpler graphs with each node defined by one element of the compound image, for example, a boy. Thus, sequences of images can be viewed as simultaneous traversals on these two simpler graphs such that, for example, the sequence in the bottom of Fig. 1*C* reflects a simultaneous traversal of A’->B’->C’->D’->A’… in the green graph factor and 7’->8’->...->12’->7’... in the black graph factor, as depicted in Fig. 1 *B*, *Right* column. Note that the incidental spatial relationships between the two features of each compound stimulus was constant throughout the experiment and are not task relevant. Thus, spatial arrangement in each compound image conveyed no information about the temporal sequences or about which feature belonged to which subprocess/graph component. We use the term “trial” throughout the manuscript to describe one sequence of 12 stimuli experienced by participants. Over the course of the experiment, participants observed multiple instances of these 12 item sequences, or trials.

To test whether people extract, and reuse, subprocess dynamics we employed a between-subjects design with two task conditions. In a prior learning phase (Fig. 1 *B*, *Left* column; behavioral results in *SI Appendix*, Fig. S2), participants in a 4-cycle prior condition experienced sequences generated from the product of a 4-state cycle graph factor and a 6-state path-graph factor (Fig. 1 *B*, *Top*-*Left*). Similarly, participants in a 6-cycle prior condition saw compound image sequences produced by combining a 4-state path-graph factor and a 6-state cycle graph factor (Fig. 1 *B*, *Bottom*-*Left*). This prior learning phase was followed by a transfer learning phase, identical for both conditions (Fig. 1 *B*, *Right* column), involving 10 entirely novel features. The latter composed 12 entirely novel compound images that were the product of a 4-state cyclic graph factor and a 6-state cyclic graph factor respectively. This between group design meant that, for each participant, only one of the two experienced abstract graph factors was common to both prior and transfer learning (e.g., in the 4-state cycle prior condition, the 4-state cycle was present in both prior and transfer learning).

The task design was such that the unfactored graphs (“product graphs”), in both task phases (i.e., prior and transfer learning), each had two disconnected subgraphs of 12 states (Fig. 1*C* illustrates this for the transfer learning product graph). These disconnected subgraphs reflect the fact that a given state in the 4-state graph factor was only combined with three of the six possible states in the 6-state graph factor during sequence presentation (e.g., state A’—the boy—was only combined with states 7’, 9’, 11’—the ball, the sandwich, and the beach—but not with states 8’, 10’, 12’—the hammer, the butterfly, and the dog). This meant during each sequence presentation, participants only experienced compound images from one of the subgraphs of the unfactored graphs (Fig. 1 *C*, *Top*). Consequently, subjects only saw transitions between 12 out of a possible full set of 24 compound images (the same subgraph, henceforth the “experienced subgraph” was used for all sequences). The remaining 12 compound images were retained as a set of held-out compound stimuli (Fig. 1 *C*, *Bottom*, inferred subgraph). These held-out compound stimuli were produced by combining a given state in the 4-state graph factor with the remaining three states in the 6-state graph factor that had never been shown together during sequence presentation (e.g., state A’—the boy—was combined with states 8’, 10’, 12’ states—the hammer, the butterfly, and the dog). The motivation for this design feature was that it provides a set of held-out compound stimuli that allowed us to test knowledge of unobserved transitions between compound stimuli. We leveraged these held-out stimuli for each task phase (i.e., prior and transfer learning phase) to assess knowledge and use of graph factorization—i.e., knowledge of the individual graph factors producing the experienced compound sequence (Fig. 1*B*). While we use the term “transfer learning” in reference to reuse of experienced subprocess dynamics throughout the manuscript, it is important to note that the experiment also implies a second type of knowledge generalization. To successfully answer inference probes, participants could generalize their knowledge about experienced transitions (as tested by experience probes) to make inferences about never experienced transitions on the graph factors (as tested by inference probes).

Following each presented sequence, across both prior and transfer learning, “experience probes” tasked participants to predict upcoming states that followed one of the compound images from the experienced subgraph (Fig. 1 *E*, *Left* column). Additionally, in both prior and transfer learning, “inference probes’’ (Fig. 1 *E*, *Right* column) presented immediately following each experience probe tested participants’ ability to predict transitions in the held out, “inferred,” subgraph. For both the experience and inference probes, this entailed participants choosing between a correct next compound image and a lure compound image. The lure compound image always matched the correct compound image with respect to one of component individual images (e.g., D’8’ -> A’9’ or C’9’), allowing us to test knowledge for each of the graph factors separately (here, the knowledge that in the 4-state graph factor component D’ is followed by A’ rather than C’). Thus, each unique compound stimulus in the sequence (and each novel compound) was used exclusively to assess knowledge of one of the two graph factors, but not both. This was intended to control for the possibility that participants could guess and remember the correct answer for each probe question by observing the same correct option repeatedly presented alongside different, changing, lure images—allowing for an inference that the consistent option must be the correct one without knowing anything about the compound sequence, or correctly inferring transitions between novel compounds.

### Humans Are Sensitive to the Feature Structure of Compound Stimuli.

**Fig. 2.**
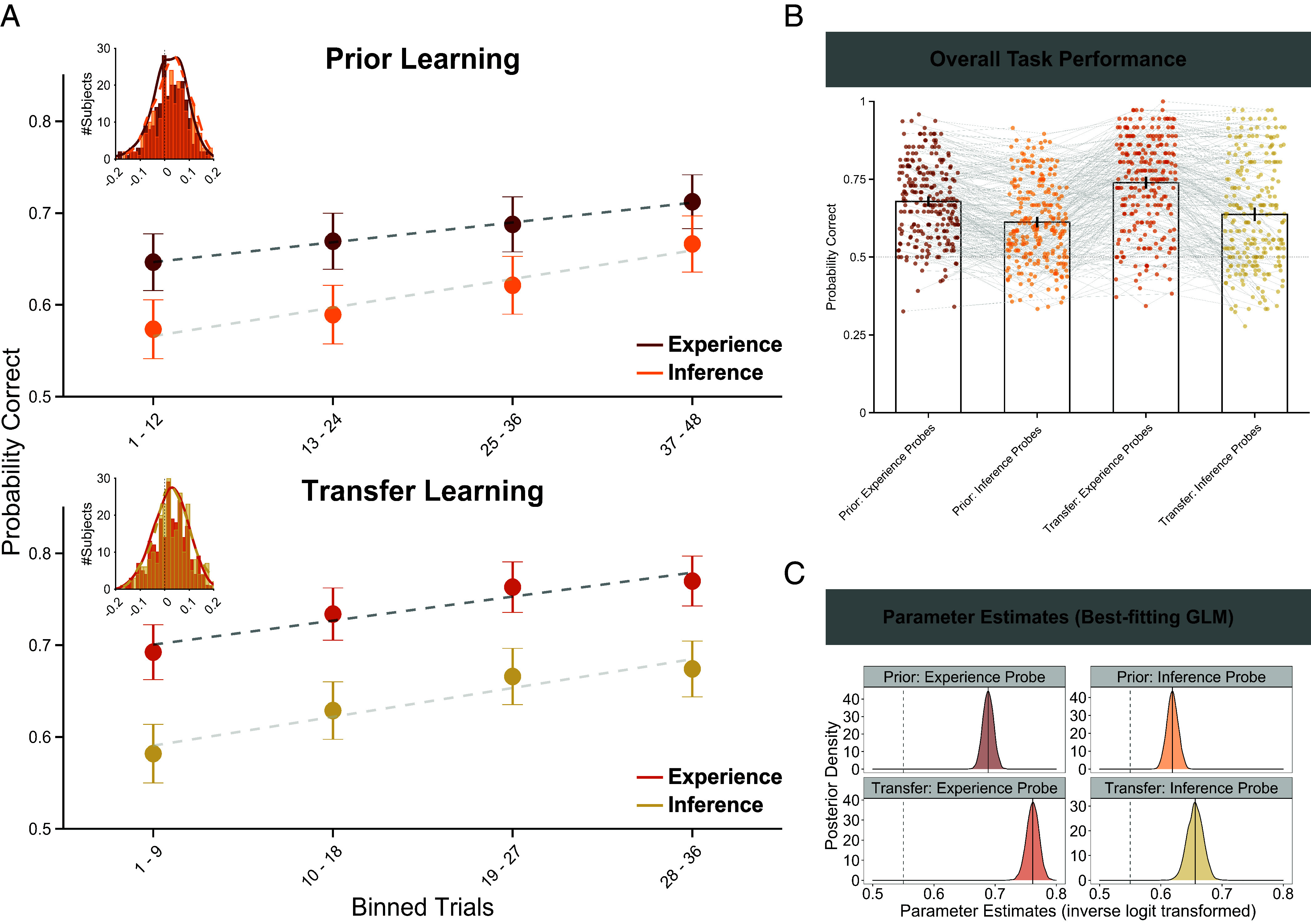
Overall task performance. (*A*) Average probabilities of correct answers across the entire sample during prior learning (*Top*) and during transfer learning (*Bottom*) in both experience probes (dark red and red dots, *Top* and *Bottom* panel, respectively) and inference probes (orange- and cream-colored dots, *Top* and *Bottom* panel, respectively) averaged in four bins of trials (12 and 9 trials per bin for prior and transfer learning, respectively). Data were averaged across probed sizes (4-state graph factor or 6-state graph factor) within each participant. Dots indicate the mean, error bars around the dots denote the SE of the mean. Dashed gray lines represent linear trends over time. Figure *Insets* show histograms of linear temporal trends over time parameter estimates, separately for experience and inference probes per learning phase. (*B*) Aggregated probability of correct answers during prior and transfer learning phase, separately for experience and inference probes. Each dot quartet connected by a solid gray line within prior and transfer learning represents one participant’s performance, bars represent group-level arithmetic means of the distribution, error bars depict the 95% CI around the mean, the dashed gray line represents the chance level point estimate (probability correct = 0.5). (*C*) Posterior density plots for each of the inverse logit transformed parameter estimates (parameter estimates on probability scale) for the probability of correct answers from the best-fitting model. Black lines denote the mean parameter estimate (posterior mean), dashed lines show the upper bound of a region of practical equivalence (ROPE, probability correct = [0.45; 0.55]).

We first verified participants learned the experienced compound sequences. In prior learning experience probes, across trials the probability of giving a correct answer increased (Fig. 2 *A*, *Top*, dark red dots), confirming that on average participants’ performance improved over time with respect to answering probe questions regarding sequences they actually experienced (linear trend coefficient: 0.02, 95%-CI = [0.019; 0.024], corresponding to a 0.21% increase of choice accuracy per trial). While the overall trend suggests effective extraction of the stimuli, a number of subjects showed negative or close to 0 learning effects over time, indicating a performance heterogeneity across the sample (Fig. 2 *A*, figure *Insets*).

We used sampling-based Bayesian inference to estimate the posterior distribution of the parameters for generalized linear models (GLM) modeling the probability of correct answers. The best-fitting GLM (*SI Appendix*, *Supplementary Materials and Methods* for model comparison) indicated the average probability of correct answers was greater than a chance level point estimate (probability correct = 0.50) for experience probes in prior learning (inverse-logit transformed intercept parameter: µ_Exp,prior_ = 0.69; 89%-Highest Posterior Density Interval (HPDI) = [0.67; 0.70], Fig. 2*B*, first bar; Fig. 2 *C*, *Top Left*). We additionally quantified the overlap between the HPDI and an a priori specified ROPE, i.e., an interval of parameter values that can be considered of negligible magnitude (“practically no effect,” probability correct = [0.45; 0.55]). There was 0% overlap between HPDI and ROPE (Fig. 2 *C*, *Top Left*), suggesting that performance greatly exceeded chance level (see behavioral performance split up by graph factor in *SI Appendix*, Fig. S2). Similar findings were obtained at a transfer learning phase (Fig. 2 *A*, *Bottom*, red- and cream-colored dots: linear trend coefficient: experience: 0.03, CI = [0.010; 0.042], corresponding to a 0.31% increase of choice accuracy per trial; parameter estimates for average performance were greater than chance-level and there was 0% overlap between HPDI and ROPE (inverse-logit transformed intercept parameter: Experience: µ_Exp,transfer_ = 0.76, HPDI = [0.75; 0.78], Fig. 2*B*, third bar, Fig. 2 *C*, *Bottom Left*). This suggests that participants also showed improvement (though not mastery) in learning new sequences experienced during the transfer learning phase.

While the above results suggest successful learning of stimulus relations, they provide little insight into a question of whether participants learned associations between holistic compound images or used more sophisticated representations (e.g., decomposed the images into features). To arbitrate between these two possibilities, we next tested performance with respect to the inference probes in prior and transfer learning phases. The compound images featured in inference probes were not presented during the observed sequences, nor could knowledge regarding transitions pertaining to inference probes be inferred based on integrating information across probe questions (*SI Appendix*, *Supplementary Materials and Methods*). Therefore, under the null hypothesis that participants only learn about compounds, accuracy would be expected to be 50% during inference probe questions. We observed a positive linear trend in probability of giving a correct answer across trials (linear trend coefficient: 0.03, CI = [0.018; 0.045], corresponding to an 0.34% increase of choice accuracy per trial) (Fig. 2 *A*, *Top*, orange dots). Overall inference probe performance was above chance-level (Fig. 2*B*, second bar, inverse-logit transformed intercept parameter: µ_Inf,prior_ = 0.62, HPDI = [0.60; 0.63]), and we observed 0% overlap between HPDI and ROPE (Fig. 2 *C*, *Top Right*). Similar findings were obtained for transfer learning (linear trend coefficient: 0.03, CI = [0.013; 0.050], corresponding to a 0.44% increase of choice accuracy per trial; there was 0% overlap between HPDI and ROPE (inverse-logit transformed intercept parameter: µ_Inf,transfer_ = 0.66, HPDI = [0.64; 0.68], Fig. 2*B*, fourth bar; Fig. 2 *C*, *Bottom Right*). These findings speak against a possibility that participants were relying solely on “compound-level” representations. Instead, they suggest that participants decomposed compound images into their component features and learned dynamic relationships between these features.

Finally, average performance during transfer learning was higher than during prior learning for both experience and inference probes (Fig. 2 *A* and *B*): no overlap between HPDI_Exp,prior_ = [0.67; 0.70] and HPDI_Exp,transfer_ = [0.75; 0.78], nor between HPDI_Inf,prior_ = [0.60; 0.63] and HPDI_Inf,transfer_ = [0.64; 0.68] (Fig. 2*C*). Similarly, the HPDI for the difference between the average probability of correct answers did not contain 0 (mean difference of 10,000 posterior samples, HPDI_Exp-diff_ = [0.06; 0.09], HPDI_Inf-diff_ = [0.02; 0.06]), suggesting increased performance in transfer learning relative to prior learning. This pattern may reflect general practice effects, or reuse of knowledge acquired in prior learning in the transfer learning phase—a question we turn to next.

### Abstracting and Compositionally Reusing Subprocesses.

We next addressed a deeper question of whether people extract, and compositionally reuse, subprocesses of experienced dynamics in new situations? To repeat the example from above, if humans discover that their experience during a home cooking evening is governed both by observing the TV chef’s cooking routine and hearing changing radio programs, can they generalize such knowledge to new situations, for example correctly applying the necessary steps involved in cooking an entirely different meal at a friend’s house while having a chat about their day at work? Thus, we next examined performance during transfer learning—when participants are confronted with entirely novel stimuli produced by traversals on graph structures that were only partially identical with those encountered during prior learning.

**Fig. 3.**
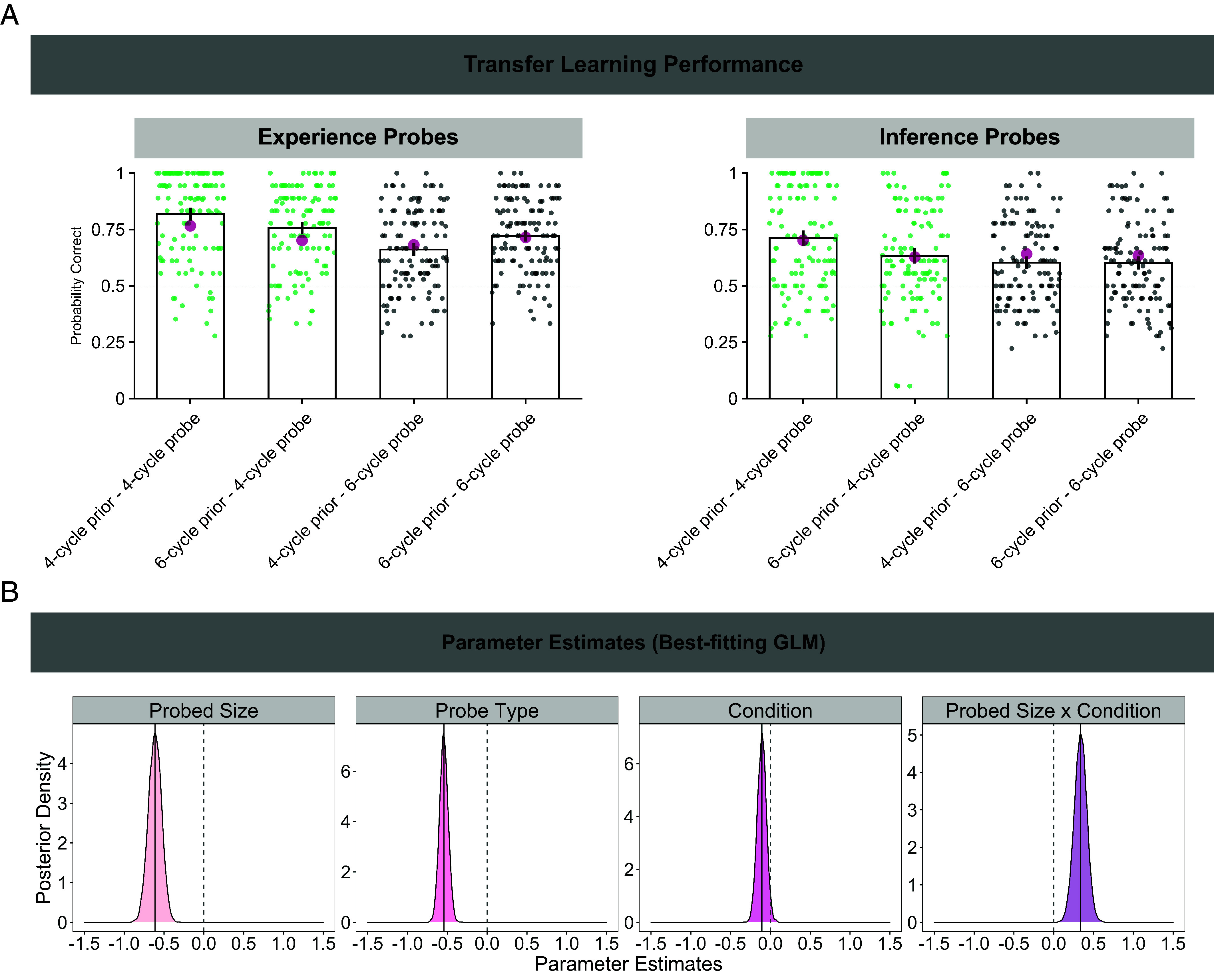
Transfer learning performance. (*A*) Aggregated probabilities of correct answers during the transfer learning phase, separately for both experience (*Left*) and inference probes (*Right*) as a function of probed size [4-state cycle (green) vs. 6-state cycle (black)] and prior learning condition (4-state cycle prior vs. 6-state cycle prior). Each dot represents one participant, bars represent the arithmetic mean of the distribution, error bars depict the 95% CI around the mean, large magenta dots represent arithmetic means of 1,000 posterior model simulations of the best-fitting model (M3, 2α/2τ) and the dashed gray line represents chance level point estimate (probability correct = 0.5). (*B*) Posterior density plots for each parameter estimate (posterior mean = black line) for the best-fitting GLM. The dashed vertical line represents a zero effect. In probed size and probe type effects, parameter estimates below zero indicate higher accuracy in the 4-cycle probes (vs. 6-cycle probes) and experience probes (vs. inference probes), respectively. In the condition effect, parameter estimates below zero indicate higher accuracy in the 4-cycle prior condition (vs. 6-cycle prior condition). The positive interaction effect indicates selectively higher accuracy in 4-cycle probes in the 4-cycle prior condition, and higher accuracy in 6-cycle probes in the 6-cycle prior condition.

We predicted that if individuals abstract experienced subprocesses (i.e., graph factors) during prior learning and reuse this knowledge during transfer learning, then they should benefit from a recurrence of this previously experienced subprocess (during prior learning). Specifically, during transfer learning, 4-cycle prior condition subjects should perform better than the 6-cycle prior condition on 4-cycle probes, and vice versa for the 6-cycle prior condition subjects. In other words, we expect a disordinal interaction effect between condition and probed size on accuracy. Alternatively, if during the transfer learning stage participants do not reuse prior knowledge (effectively treating it as a new session), there should be no interaction. In effect, we ask whether the probability of being correct during transfer learning varies as a function of prior learning condition (4-cycle prior condition vs. 6-cycle prior condition) and probed size (during transfer learning: probes testing knowledge of 4-state graph factor vs. 6-state graph factor) (Fig. 3*A*).

We defined four Bayesian multilevel GLMs (GLM3–GLM6, *SI Appendix*, Eqs. **S3**–**S6**), modeling different processes that might have generated the observed behavioral data during transfer learning. Correct responses to experience and inference probes were analyzed in a joint model, so as to reduce the number of tests of the same statistical hypothesis and to increase statistical power. The most parsimonious model (GLM5; *SI Appendix*, *Supplementary Materials and Methods* for model comparison) featured a nonzero interaction effect between probed size (i.e., 4- vs. 6-cycle probe questions) and prior learning condition (interaction effect: µ_SIZExCOND_ = 0.33, credible interval (CI) = [0.21; 0.46], Fig. 3*B*, fourth panel), but no three-way interaction effect differentiating an interaction effect by probe type (experience and inference probes). Disentangling the probed size x condition interaction effect using post hoc contrasts, we found that the interaction was disordinal: average accuracy was higher for 4-cycle probes in the 4-cycle prior condition (vs. 6-cycle prior condition; Fig. 3*A*, green dots; mean difference of 10,000 posterior samples: 0.35, CI = [0.27; 0.43]) whereas for 6-cycle probes, performance was superior in the 6-state cycle prior condition (vs. 4-cycle prior condition; Fig. 3*A*, black dots; mean difference of 10,000 posterior samples: 0.12, CI = [0.05; 0.19]). The nonoverlapping CIs between posterior mean differences suggest that performance differences for the 6-cycle were less pronounced than for the 4-cycle graph factor. Posterior distributions for the other parameter estimates of the best-fitting GLM (probed size, probe type, condition) are depicted in Fig. 3*B*. Note, highly similar results were observed in a smaller, independent, sample of healthy human volunteers (*N* = 137, online sample, *SI Appendix*, Fig. S1).

The 6-cycle inference probe condition difference was qualitatively less pronounced than was the case for experience probes (Fig. 3*A*). Nevertheless, the behavioral data were best accounted for by GLM5, a model that did not feature a three-way interaction effect differentiating the probe size × condition interaction effect by probe type (experience and inference probes). This suggests absence of model evidence that the probed size × condition interaction effect differs across probe types. Interestingly, a qualitatively lower 6-cycle inference probe condition difference effect was also observed in posterior simulations using successor feature models (*Computational Modeling*). We discuss additional potential explanations for these qualitative differences based on capacity limitations and explicit knowledge in *SI Appendix*, Fig. S4 and *Supplementary Information Text*.

The observed pattern of results is not accounted for by general practice or nonspecific learning effects (e.g., developing strategies for how to learn sequences—”meta-learning”), as such accounts predict similar performance for both probed sizes across conditions. Instead, the results support a conclusion that during prior learning participants extracted subprocess dynamics that produce their experience, and reuse dynamics during transfer learning that were consistent across both task phases consistent with reuse of elements from prior experience in a compositional manner.

Further support for this notion was provided by a finding that the benefits in re-encountering a previously experienced subprocess manifest in the very first trials of the transfer learning phase (*SI Appendix*, Fig. S3 and *Supplementary Information Text*).

### Computational Modeling.

**Fig. 4.**
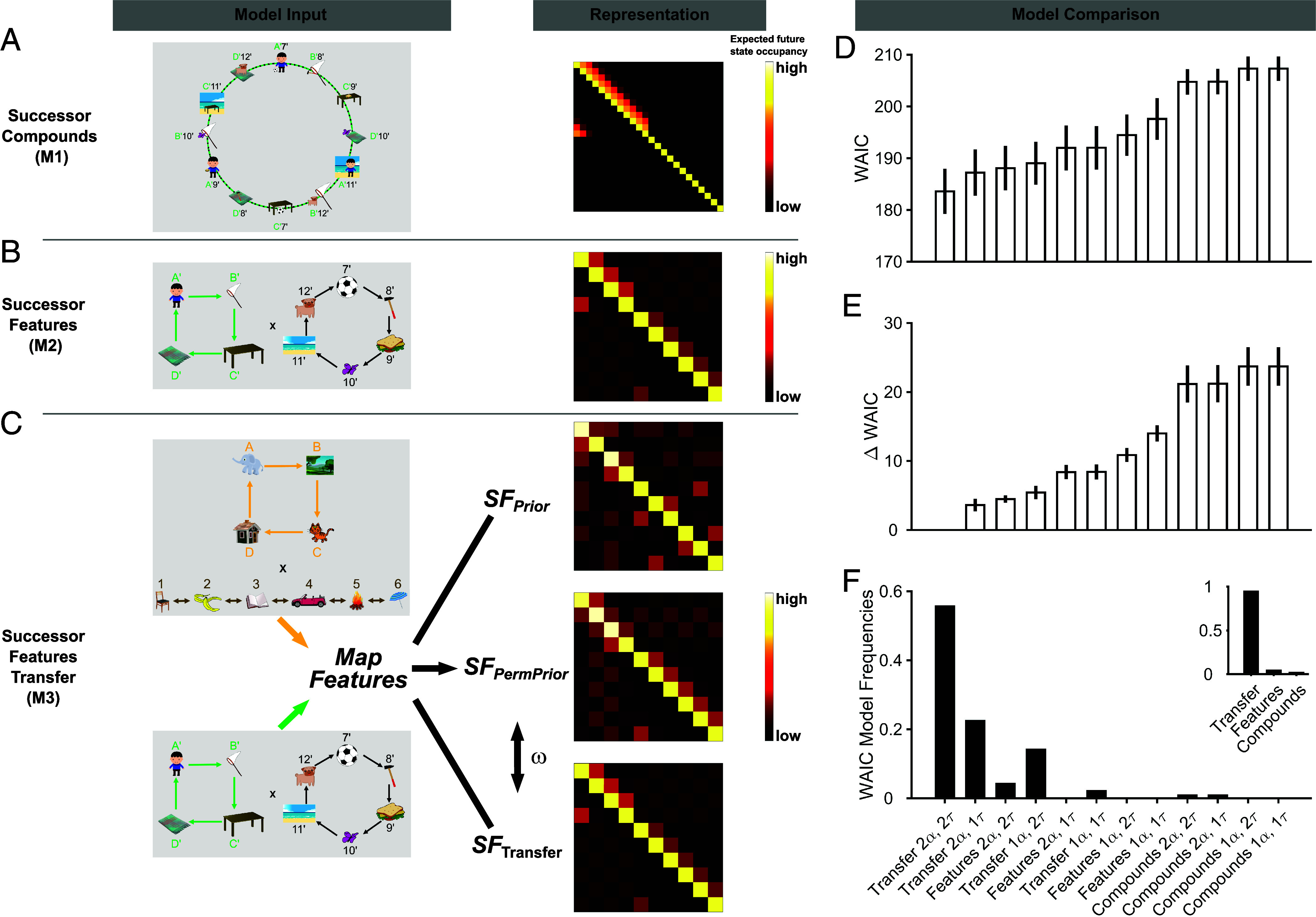
Computational models and model comparison results. (*A*) A successor compound model (M1) learned an expected future state occupancy from the sequence of unique compound states and stored this quantity in a successor compound matrix (Representation column). Each row and column represent one compound state. For illustrative purposes, we depict the expected, discounted future compound occupancy after the completion of all trials in transfer learning only. *Top* rows and leftward columns correspond to experience compounds whereas *Bottom* rows/rightward columns correspond to inference compounds. Note that because inference states are never experienced during sequence learning they include 0 as nondiagonal successor entries. (*B*) A successor feature model (M2) learned expectations about the sequences of features in each graph factor separately (same updating mechanism as M1, but operated over two separate features, for the 4-state and 6-state factor separately). For illustrative purposes, we depict the expected, discounted future feature occupancy after the completion of all trials in transfer learning only. Each row and column represent one feature. The 4 topmost rows and leftmost columns represent features associated with the 4-cycle factor whereas remaining rows/columns represent features of the 6-cycle factor. Note that because this model does not realize that features are arranged into two separate sequences, nondiagonal blocks contribute nonzero successor entries. (*C*) The successor feature transfer model (M3), uses the same updating mechanisms as M2 but, additionally, incorporates transfer learning abilities. M3 leverages a permutation algorithm that searches for the optimal mapping between states/features encountered during prior learning and those observed in transfer learning to map prior knowledge onto new experiences. The permuted prior learning successor feature representation and newly acquired transfer learning successor feature representation are weighted by a weighting parameter ⍵ to enable experience and inference probe choices. (*D*) Average WAIC values for each model. Per model, 4 model variants with different parameterizations were fitted and compared: 1⍺/2⍺, 1τ/2τ. (*E*) WAIC value differences relative to the best-fitting model (2⍺/2τ variant of M3). In (*D*) and (*E*) bars represent the mean, error bars are the 95% CI around the mean. (*F*) WAIC model frequencies, normalized counts of how often a respective model had the lowest WAIC for individual participants. The figure *Inset* shows the aggregated frequencies across the four variants of each model class (Transfer, Features, Compounds).

To develop a more in-depth account of our data we devised three computational models (Fig. 4). The models were all derived from the class of successor predictive models (43, 44, 47), which broadly rely on learning the temporal associations between stimuli in experienced sequences. For instance, these models learn how compound images, or the individual features within these compounds, are interconnected. Thus, models M1-3 updated a successor representation matrix. We provide an overview of the models here, with full details and formalization available in *SI Appendix*, *Supplementary Materials and Methods* section.

The first model (M1), termed the successor compound model (43, 44, 47) (*SI Appendix*, Eqs. **S7** and **S8** and Fig. 4*A*), demonstrated a limited understanding of the stimulus structure. It treated each compound image encountered in the sequences as a unified whole, failing to recognize that the observed compounds consist of two distinct features. Consequently, for each experienced compound M1 learned their expected future occupancy— essentially, the expected number of future observations of all compounds associated with a specific compound.

The second model (M2), the successor feature model (42) (*SI Appendix*, Eqs. **S9** and **S10** and Fig. 4*B*), deviated from M1 by breaking down compounds into corresponding pairs of individual features. However, this model was not imbued with knowledge that these features were tied to a decomposition of two distinct sequences of features. As a result, M2 learned temporal relationships between features that comprised each compound image (i.e., the expected future feature observations of all features given particular features). Importantly, this model learned a dedicated successor feature matrix for each phase (prior and transfer learning), without integrating knowledge between these phases.

The third model (M3) was a newly developed extension of a successor feature model, extending it by addition of a computational mechanism supporting transfer learning abilities (Fig. 4*C*). As such, M3 integrated information learned during the prior learning phase with information acquired during transfer learning. This reuse of prior knowledge was enabled by finding an optimal one-to-one mapping between states/features encountered during prior learning and those observed in transfer learning (*SI Appendix*, Eq. **S11**). This mapping maximized the similarity between the successor representation matrices of both phases and can be conceptualized as a structural scaffolding formed by prior experience for incoming new information (see Fig. 4*C* for a graphical depiction; *SI Appendix*, *Supplementary Materials and Methods* for details). For example, if the elephant was followed by a landscape image during prior learning, M3 leverages this knowledge to infer that, during transfer learning, a feature corresponding to the elephant (e.g., the boy) would be followed by a feature corresponding to the landscape image (e.g., a net). The “remapped” prior learning successor feature representation and newly acquired transfer learning successor feature representation were mixed to enable experience and inference probe choices using a weighting parameter ⍵—allowing us to quantify an individually varying strength of prior knowledge reuse across participants (*SI Appendix*, Eq. **S12**). For each of the above models, we implemented four different variants obtained by factoring the number of learning rates (1 overall ⍺ parameter or 2 separate ⍺ parameters for prior and transfer learning phase) and the Softmax stochasticity used to generate choices (1 overall τ parameter or 2 separate τ parameters for experience and inference probes). Thus, the overall model space contained a total of 12 different computational models.

### A Successor Predictive Model Reusing Prior Knowledge Recapitulates Task Behavior.

We leveraged apriori simulations to provide an intuition for the behavioral patterns predicted by each of the three models and then selected those models that were, in principle, capable of capturing subjects’ behavior (*SI Appendix*, Fig. S5 and *Supplementary Results*). To this end, we simulated 250 model instantiations per computational model with fixed parameters (*SI Appendix*, *Supplementary Materials and Methods* for details), ensuring matched parameters across prior learning conditions. These apriori simulations indicated that the three candidate models, incorporating different assumptions about the underlying principles of the learning process, made highly distinct behavioral predictions that could be differentiated (*SI Appendix*, Fig. S5).

We next fit the selected models to observed behavioral data to implement a quantitative model comparison, including quantifying the relative model frequencies in our sample. We fit the computational models individually to each participant’s observed behavioral data to obtain maximum likelihood estimates (*SI Appendix*, Eq. **S13**) for each model’s free parameters (*SI Appendix*, *Supplementary Materials and Methods* for details, distributions of best fitting parameter estimates per model depicted in *SI Appendix*, Fig. S6). The model comparison was conducted using the Widely Applicable Information Criterion (WAIC), a model comparison metric that approximates a model’s leave-one-out cross-validated predictive accuracy. WAIC values indicated that the 2⍺/2τ variant of M3 best fit the behavior of participants (Fig. 4*D*, WAIC value differences relative to best-fitting model in Fig. 4*E*). Quantifying the percentage of participants for which each candidate model fit the behavioral data best indicated that the 2⍺/2τ variant of M3 was also the most frequent model in the sample (55.6%, Fig. 4*F*). For 94.2% of participants, any variant of transfer models fit their behavioral data best (Fig. 4 *F*, *Inset*), indicating that the behavior of the vast majority of the sample was best explained by predictions of successor feature models with transfer learning abilities (M3).

The above metrics provide insights into the prevalence of different candidate mechanisms approximating human behavior among participants. However, we consider a crucial test in differentiating candidate models, as providing a meaningful explanation for actual observed behavior, involves assessing each models’ ability to account for observed behavior in posterior simulations (48). These posterior simulations allow for model falsification, as they address whether a model can reproduce a specific data feature (e.g., a behavioral effect) of interest, using the best-fitting parameters values optimized using the observed data. We applied maximum likelihood parameter estimates for each participant to simulate choices for the presented probe questions and calculated the accuracy rates of simulated choices (as with observed choices). The data feature of interest used to falsify the candidate models was the disordinal interaction effect of probed size x condition during transfer learning—with higher performance on the 4-cycle graph factor and lower performance on the 6-cycle graph factor for the 4-cycle prior condition (relative to the 6-cycle prior condition). To visualize this effect, for both data and simulations, we computed the average across-condition difference between the accuracy for each probed size (4-cycle and 6-cycle probes), separately for each probe type (experience and inference probes; *SI Appendix*, Fig. S7*A*, reproduced four times for convenience).

The 2⍺/2τ variant of M3, that best fit the behavioral data, also recapitulated the data feature of interest in posterior simulations, showing a higher average probability of being correct on 4-cycle probe questions for the 4-cycle prior condition than for the 6-cycle prior condition, and, a lower average correct probability on 6-cycle probe questions for the 4-cycle prior condition than for the 6-cycle prior condition, across both experience and inference probes (*SI Appendix*, Fig. S7*D*, fourth panel; Fig. 3*A* large magenta dots). This is because M3 accurately maps states/features that were consistently encountered during prior learning to those observed in transfer learning (e.g., the 4-state cycle in the 4-cycle prior condition), allowing a generalization of knowledge acquired during prior learning to infer the structure of corresponding components in transfer learning. In posterior simulations, using the successor predictive models without transfer learning abilities, neither M1 (*SI Appendix*, Fig. S7*B*), nor M2 (*SI Appendix*, Fig. S7*C*) recapitulated the above behavioral data features. Crucially, there was no resemblance to a positive difference in 4-cycle probes, and a negative difference in 6-cycle probes, as observed in the data. These results suggest that M1 and M2 are unable to account for specific effects of interest and should be rejected in explaining the observed data. However, since the 2⍺/2τ variant of M3 could reproduce the behavioral effects of interest sufficiently well, we conclude that it provides a highly plausible candidate mechanism for explaining the observed behavior.

## Discussion

The dynamics of real-world situations invariably involves separable subprocesses, raising a question as to how humans manage everyday situations that reflect such complex dynamics. In this paper, we examine a candidate mechanism—compositional generalization of knowledge regarding subprocess dynamics—as an explanation of how we generalize knowledge from complex everyday experiences. We provide evidence that humans parse subprocesses that generate the dynamics of experienced situations. More importantly, we show they abstract these subprocesses away from the specifics of their sensory experience to apply them flexibly in novel situations. Our behavioral findings indicate that subjects form separate representations of both the generative process and the stimuli producing their experience, in line with previous findings (2, 39, 41, 49–51). Notably, our study extends on previous cognitive-computational accounts of structural inference in state spaces by providing evidence for abstraction of subprocesses underlying experience, beyond the specificity of encountered stimuli. We suggest that such a form of knowledge representation in memory facilitates abstraction over concrete events and, as we show here, enables reuse of abstracted subprocesses in an efficient and compositional manner to support generalization of knowledge to novel, never experienced, situations.

Two key empirical findings support the notion of compositional generalization of acquired abstracted knowledge. First, during transfer learning participants made more accurate predictions about entirely new stimuli solely on the basis of having previously encountered similar subprocesses governing the relationships between other stimuli. This effect was seen despite sequences being produced by a novel composition of structural forms that only partially overlapped with previous experience. Second, already during the very first trials of transfer learning we observed performance advantages specific to subprocesses shared across prior and transfer learning. This rapid form of adaptation is one not expected under models forming predictive representations exclusively from exposure to stimulus sequences, where these ignore knowledge representations extracted from previous experiences. In other words, such models would not predict performance differences contingent on a structural consistency across prior and transfer learning—either after limited exposure or across the whole task. Instead, the behavioral findings support a notion that humans form representations of encountered subprocess dynamics to enable compositional reuse and rapid adaptation in novel contexts. While this reuse is limited to the case of complete structural overlap with previous experiences when employing conjunctive representations, a factorized representation affords a plurality of new ways to create complex dynamics as explanations for experiences.

We did not instruct participants that the task would consist of two dependent phases, whereby knowledge acquired during an initial task phase would, in a second phase, bear relevance to a new learning environment. Thus, the behavioral results favor an interpretation that a factorized knowledge representation is formed automatically, based on cognitive-computational advantages, enabling an economical recombination of subprocesses that comprised building blocks of their experience (e.g., a compound representation of our product graphs consists of 6 × 4 = 24 states, whereas a factorized representation entails a lower number of states, 6 + 4 = 10). As such, a factorized representation allows reuse of computations, facilitates compositional knowledge generalization, and enables rapid inferences across spatial and abstract domains (2). In this regard, our findings provide an instance of compositional generalization based on reuse of entire structures that match, in size and form, in a new combinatorial context. This can be considered a subclass of a broader understanding of compositional generalization that proposes selection and reuse of structural primitives from a larger set of such primitives that can be flexibly adapted to novel situations (e.g., by changing the size of the state space or by applying it to different tasks/contexts).

Our behavioral results can also be interpreted by reference to structure-mapping theory, which proposes a mapping of knowledge representations across domains dependent solely on syntactic properties or relations between objects, irrespective of the content or actual object-attributes (45, 46). The study extends on these theoretical accounts in showing that humans can exploit such structure-mapping even in more complex environments characterized by multiple dynamical subprocesses. Crucially, the structure-mapping between subprocesses seen in our study appears highly selective to subprocesses that exhibit high structural similarity, suggesting that transfer learning is facilitated for instances involving a matched state space size, and structural form, between training and transfer [which putatively simplifies structural mapping, but see ref. 52 (chapter 6, experiments 1 and 2) for discussions on generalizations of the same structural form but different state space sizes]. Future studies, by employing additional control conditions (e.g., a prior learning condition including no cycles), could determine whether there is positive or negative transfer between cycles of different size (e.g., whether learning a cycle of size 4 facilitates or inhibits, compared to control, learning of a size 6 cycle in transfer learning).

Related research on ordinal list learning indicates agents not only form item–item associations, but also represent ordinal position information of list items (10–16). This knowledge facilitates efficient learning of novel lists wherein items retain their originally learned position. We extend these findings in several important ways. In ordinal list learning tasks participants are presented at each moment with a single structure (i.e., an ordinal list), and hence do not address the challenge of component factorization. Additionally, in a list learning paradigm participants extract abstract item-specific knowledge (e.g., how many items precede or follow the current item) which allows a rearrangement of the same items in novel mental representations. By contrast, in our task participants extract abstract structural information away from the identity of specific items, and these structural forms can subsequently be generalized to novel situations.

We falsified model-based behavioral predictions derived from apriori simulations, and posterior simulations applying optimized parameters in successor predictive models without transfer learning abilities (M1 and M2), by their failure to capture the observed transfer learning behavior (48). The successor compound model (M1) failed to capture an above chance-level human inference probe behavior (*SI Appendix*, Fig. S4*D*), while the successor feature model (M2) could not recapitulate the key behavioral effect of interest, namely condition-specific performance differences for graph factors consistent across prior and transfer learning. This inability to account for the data is likely to reflect the fact that these successor predictive models form temporal associations solely between compound images/features. In other words, successor predictive model architectures do not allow a transfer of knowledge and its reuse in new contexts.

We found subjects’ behavioral data was consistent with predictions of a newly developed successor predictive model that reuses prior learning representations during transfer learning (M3). This model architecture suggests a computational mechanism for transfer of learned representations across contexts. Reuse of prior knowledge is enabled by finding an optimal one-to-one mapping between features encountered during prior learning and those observed during transfer learning. These remapped features can be conceptualized as a structural scaffolding formed by prior experience for incoming new information—allowing a boosting of prediction accuracy specifically for subprocesses consistently experienced across distinct task phases. Notably, the successor feature model with transfer learning abilities reproduced the interaction effect of probed size and condition sufficiently well in posterior simulations, suggesting it is a plausible candidate mechanism to explain observed behavior. We acknowledge that M3 represents an extension of previous successor predictive architectures, and our data show that endowing successor predictive models with a purely data-driven mechanism to map prior knowledge representations to novel experiences can yield results resembling actual human behavior. This insight is important given successor predictive models have struggled to explain human generalization and transfer learning abilities (41). While M3 was an extension of successor predictive models, the model and our behavioral task take inspiration from ideas proposed within previous modeling architectures related both to a cognitive map framework (2, 39, 41). We also acknowledge structural inference models (53), that combine inferences over relational structures for objects and features (54), forming an abstraction to generalize knowledge (52) (chapter 6, experiments 1 and 2) or perform recursive operations over hierarchies [fragment grammars (33, 34)]. A common idea within the latter and our models is that components underlying experience can be abstracted away from sensory details to form reusable structural units. This enables previously acquired knowledge and learned rules to be reused in new situations. While previous accounts focused on the case of inferences over a single structural form, our model extends to generalization and reuse of multiple, simultaneously experienced subprocesses in new contexts, where only a subset of the learned rules/relations is relevant.

For the successor predictive model with transfer learning abilities, we propose a mechanism involving an optimal mapping between prior learning and transfer learning task features, allowing boosted performance for predictions over structures consistently experienced across these task phases. This feature-mapping mechanism was not designed with a specific biologically plausible neural mechanism in mind, and makes no claim about underlying neurobiology. However, we note prior computational and empirical work related to the hippocampal formation and entorhinal cortex propose that grid cells may function as a basis set of representations for different structural forms (2, 27, 39, 41), and these could provide a structural scaffolding for new experiences. One simplifying aspect of our experimental task was the equal number of prior learning and transfer learning features (allowing for relatively simple one-to-one mapping). Real-life learning scenarios, however, often necessitates finding (structural) similarities between environments of different sizes. It is plausible that grid cell basis set representations may be leveraged to extend feature mappings from the special case of equal feature numbers to the more general (and realistic) case of an asymmetry in the number of features across environments. In cases where there is no exact structural match between source and target domains (one-to-many or many-to-one mappings), hippocampus and entorhinal cortex could leverage an eigendecomposition of the prior learning phase successor representation. The resulting eigenvectors could be size-adjusted (stretched or compressed) to reflect the size of the transfer-learning environment and estimate the transfer learning successor representation (following the approach outlined by ref. 41). Thus, a pressing research question arising out of our behavioral results pertains to the precise neural implementation of the observed factorization, abstraction, and mapping of subprocesses across contexts and size-varying structures.

In the present study, we deliberately used arbitrarily selected stimuli and combined these into compound images. This could have artificially increased the salience of individual features, facilitating decomposition of compounds and assignment of features to each graph factor. In turn, this could lead to an overestimation of learning and factorization abilities. However, even in related studies combining two different artificial grammars for audio-visual integration (e.g., ref. 24), stimuli from different grammars were presented across different modalities, randomly interleaved, and separated in time. In our study, such straightforward feature separation was not enabled by experimental design, but instead participants needed to extract features of simultaneously presented compound images purely from the statistical regularities of the underlying generative subprocesses. On the other hand, the setting of the study might also have deprived participants of stimulus properties and learning strategies commonly leveraged to factorize subprocesses of experiences in real-world settings. In most naturalistic learning scenarios, unlike in the present experiment, humans often actively interact with, control and manipulate elements of experience to test hypotheses about causal relationships in the environment, something that potentially allows faster discovery and enhanced generalization of subprocesses. Additionally, in such settings, humans can draw upon rich prior knowledge about semantic relationships and category structures, as well as exploit perceptual similarity between stimuli to either rapidly separate causal factors in the environment or facilitate finding mappings between old and new situations. This prior knowledge, as well as perceptual biases, provide a natural scaffolding for factorization, abstraction, and reuse of subprocesses in novel situations. The constraints necessarily introduced by an experimental setting might explain the moderate benefits from compositional reuse observed in our task, and we suspect these may reflect a lower-bound estimate of human learning capacities in more natural settings.

A further challenge to real-life compositional reuse is that we often encounter novel situations that display structural similarity with a range of different prior experiences. To benefit from the wealth of past experiences stored in memory, we need to select and weight candidate structures, an integration process across multiple different prior experiences. While our current computational modeling work does not explicitly address these possibilities, such selection and integration could be achieved by introducing weighting parameters for different prior successor representation matrices, e.g., prior learning matrix 1, prior learning matrix 2, …, prior learning matrix *N*. These weightings could be obtained by comparing the similarity of different permuted prior learning and transfer learning matrices so as to find the closest candidate or structural match between a memory and current experiences. It is also possible that rich semantic or perceptual cues found in naturalistic learning scenarios may facilitate detecting similarities between episodes.

Beyond human learning and generalization of structured representations, models for building predictive representations of graph structured data have gained recent attention in machine learning and AI. Graph neural networks [GNN (55)] comprise deep learning–based algorithms that extract and use information from structured data sources across various applications [e.g., knowledge graphs, predicting molecules and receptor binding for drug discovery (56)]. A potential application of the transfer learning model proposed here rests in its relatively simple structure and computationally inexpensive mapping algorithm, that enables discovery of commonalities between experienced graph structures. Such neuroscience-inspired algorithms could, in principle, enhance transfer learning and generalization capabilities of GNNs between training data and novel experience.

## Materials and Methods

We summarize the participants, experimental procedures and design, behavioral analyses, and computational here. Detailed descriptions are provided in *SI Appendix*, *Supplementary Materials and Methods*).

In the study, 266 participants were recruited online via Prolific (https://www.prolific.co/), with 238 ultimately included after exclusions (N = 137 in replication sample; only including subjects reporting absence of past/present mental health conditions, absence of neurological conditions and minimum approval rates >95% from previous experiments on Prolific). Participants received an average compensation of £8.01 per hour. The study was approved by the University College London Research Ethics Committee (reference number: 6,649/004) and conducted in accordance with the Declaration of Helsinki.

Participants engaged in a sequence learning task that involved observing and predicting transitions between compound images generated according to specific subprocesses and graph structures. The experiment tested their ability to infer and predict transitions between novel image compositions, examining whether they could abstract and reuse learned subprocesses in a new context during a transfer learning phase.

Behavioral data were analyzed using several Bayesian multilevel GLMs [GLM1-6, cf. (57)] to assess participants’ performance in predicting transitions between compound stimuli (experience probes) or infer novel transitions (inference probes). Models were fitted using No-U-Turn samplers and compared using the WAIC and Pareto-Smoothed Importance Sampling.

Three successor predictive models were developed to simulate and fit participants’ learning and transfer of knowledge in the sequence learning task. The first model (M1) focused on predicting compound transitions, the second (M2) on learning feature sequences, and the third (M3) on transferring prior learning to new tasks by aligning and reusing learned structures. Model parameters were optimized using maximum likelihood estimation and compared using WAIC. Posterior simulations using the individually optimized parameters were performed to reproduce key behavioral effects.

## Supplementary Material

Appendix 01 (PDF)

## Data Availability

Behavioral and model simulation data underlying the results and figures in this paper, as well as the code are available at: https://github.com/LLuettgau/comp_generalization_PNAS (58).
