# Supplementary material for: Decomposing dynamical subprocesses for compositional generalization
Source: Proc Natl Acad Sci U S A. 2024 Nov 8;121(46):e2408134121. doi: 10.1073/pnas.2408134121 (PMC11573675; doi:10.1073/pnas.2408134121)
Supplement: Supplementary file 1 — Appendix 01 (PDF) [file pnas.2408134121.sapp.pdf]

Supporting Information for

Decomposing dynamical subprocesses for compositional generalization

Lennart Luettgau*, Tore Erdmann, Sebastijan Veselic, Kimberly L. Stachenfeld, Zeb Kurth-Nelson, Rani Moran, Raymond J. Dolan

*Corresponding author: Lennart Luettgau
Email: lennart.luettgau@gmail.com

This PDF file includes:

Supplementary Material and Methods
Supporting Information Text
Figures S1 to S7

Supplementary Materials and Methods

Participants and procedures

Participants were recruited online via Prolific (https://www.prolific.co/) and participated in the study between June 6th and 7th 2022. We included participants reporting absence of past or present mental health conditions, absence of neurological conditions and minimum approval rates >95% from previous experiments on Prolific. There were no location-based restrictions to participation.

A total of 266 participants took part in the study, with 239 submissions included in the final sample (*N* = 1 rejected due to providing a wrong completion code and finishing the study unreasonably fast, *N* = 7 for timed-out sessions, *N* = 16 did not pass an initial quiz about the experiment, *N* = 1 did not meet the training performance criterion in two attempts, *N* = 2 participants reported experiencing malfunctions during the task, e.g. long temporal lags between images, program not recording responses). We did not perform a formal power calculation to determine sample size but based the chosen number of recruited participants on sample sizes in previous online studies conducted in our lab. Participants were rewarded and received an average reward of £8.01 per hour. The study was approved by the University College London Research Ethics Committee (reference number: 6649/004) and conducted in accordance with the Declaration of Helsinki.

Upon declaring interest in participating in the study, participants were linked to Gorilla (https://app.gorilla.sc), an online experiment builder that hosted the experiment and questionnaires. Following provision of informed consent for participation, subjects then accessed written instructions regarding the experiment. Following these instructions, they were quizzed regarding knowledge of the experimental task. The quiz featured one attention check question to identify non-compliant, and inattentive, subjects who were excluded from further participation. Participants not passing the attention check question received no monetary compensation. Only participants who correctly answered all four experimental task quiz questions, as well as passed the attention check, proceeded to a training session. In cases of failure to complete the quiz, participants were allowed to re-take it one further time after once again accessing study instructions. If they failed on any quiz question for a second time, they were excluded from the study (*N* = 16) but received partial monetary compensation for the time spent (£4).

The training session comprised 10 trials to familiarize participants with the experimental setup and response modalities. This involved presenting a sequence of images unrelated to the stimuli used in the main experiment and asking subjects to make predictions about upcoming stimuli. Participants had to achieve at least 70% correct responses during training and were allowed to retake the training one further time (*N* = 5). If they did not exceed a threshold of 70 % correct responses at this second attempt, they were excluded (*N* = 1). After the training session, participants were randomly assigned to one of the two experimental conditions (see Experimental design and behavioral task). Upon completion of the experimental task, participants filled out a sociodemographic questionnaire and a post-experimental questionnaire assessing strategies used and their general task understanding. At the end of the study, participants received a debriefing about the aims and hypotheses of the study.

In total, 239 healthy volunteers were included in the sample. One participant was excluded from statistical analyses due to having >10% missed responses overall in probe questions, leaving a total of *N* = 238 participants for final analyses (median age: 28 years, *SD* = 10.61, range = 18 – 70, 139 males), *N* = 119 subjects per condition.

Experimental design and behavioral task

Participants performed a sequence learning paradigm involving presentation of sequences of image compositions. In line with our between-subjects design, during prior learning one group of participants (condition 1, 4-state cycle prior) observed sequences of image compositions (12 compound images total, composed from 10 individual features) that were drawn from the product graph (tensor product) of a cyclic graph factor with 4 states (e.g. A->B->C->D->A …) and a path-graph factor with 6 states (e.g. 1<->2<->3<->4<->5<->6, 4 x 6 factorization). The other group (condition 2, 6-state cycle prior) saw compound image sequences produced by a cyclic graph factor with 6 states (e.g., 7->8->9->10->11->12->7…, 4 x 6 factorization) and a path-graph factor with 4 states (e.g., E<->F<->G<->H). Participants observed a total of 48 image sequences during this ‘prior learning’ phase.

Subsequently, we tested all subjects during a transfer learning phase, in which they experienced sequences of entirely new image compositions (12 compound images total, composed from 10 novel individual features) generated from the product graph of two cyclic graphs (4 states, e.g. A'->B'->C'->D'->A'…, and 6 states, e.g., 7'->8'->9'->10'->11'->12'->7'…, 4 x 6 factorization). Participants observed 36 image sequences during this transfer learning. Stimuli representing states in the graphs comprised depictions of everyday objects, landscapes, and animals/humans, that could be arranged to create meaningful holistic scenes (e.g., a boy and a ball, or a cat in a car). Images were selected from Pixabay (https://pixabay.com/), with two images manually assembled to form a compound image using Inkscape (https://inkscape.org/). We used two non-overlapping and unrelated sets of ten images to compose the compound images. Assignment of sets and positions of the images used to create compound images, with respect to underlying graph structures producing the observed sequences, were counterbalanced across subjects and conditions. This mitigated a potential confound in learning, as well as choice biases, due to idiosyncrasies inherent in compound images.

The nature of the factorization, featuring graph factors with even numbers of nodes and a common multiple, meant the current position in both graph factor's transition matrices stayed in sync throughout the respective task phase – naturally allowing us to present only a subset of 12 compound stimuli. Therefore, during both task phases, participants did not experience the full set of all 24 possible image compositions implied by the 4 x 6 graph factorization. The held-out sets of 12 compound stimuli provided a context for assessing knowledge of graph factorization.

Each transfer learning sequence comprised 12 compound image presentations, in center screen (stimulus presentation duration: 1000 ms), interleaved by an inter-stimulus interval of 750 ms marked by a blank screen. Following each presented sequence, two probe questions asked participants to predict upcoming states. The first question tested knowledge of one (pseudo-randomly selected) transition of the 12 experienced transitions between observed compound stimuli in the sequence (experience probes). A second question assessed an ability to infer and predict never experienced transitions, by testing one (pseudo-randomly selected) transition of the 12 transitions between novel image compositions, where these are implied by the 4 x 6 graph factorization (inference probes). We reasoned that participants could only attain above chance predictions about transitions between the 12 never experienced image compositions if they had encoded a factorized representation of the true generative state space underlying the observed image compositions. In each probe question, participants first saw a randomly selected compound image from the experienced sequence, or an entirely new compound image, linked to a probe question "Imagine you see this image. What would be the next image?" (duration: 2500 ms). Each compound image was presented four times across prior learning, and three times across transfer learning. Following this, two compound images – the correct next image and a lure image – were presented as choice options on the left- and right-hand side next to screen center. Crucially, on each probe question, the lure image was matched with the correct option on one of the two features (e.g., A1 -> B2 or D2), a design feature that allowed us to test knowledge of both graph factors underlying the observed sequence separately, as well as assess knowledge about transitions between novel compounds.

Each unique compound stimulus in the sequence (and each novel compound) was used exclusively to assess knowledge of one of the two graph factors, but not both. This controlled for a possibility that participants could guess and remember the correct answer to each probe question by observing the same correct option repeatedly presented alongside different, changing lure images. This would allow an inference that the consistent option is the correct one – even without knowing anything about the compound sequence, or correctly inferring transitions between novel compounds. Each transition within each graph factor was probed equally often in both prior and transfer learning phase. During each probe question, participants had 4500 ms to select the left or right option by using the "x" or "m" button on their keyboard and if they failed to respond within this time window, a timeout warning was presented for 1500 ms and the probe question was coded as a missing event. We excluded participants with >10 % missing responses overall (prior and transfer learning, across both experience and inference probes). Participants did not receive feedback about whether they answered correctly or not on any given probe question. Between the experience and inference probe questions, there was a blank screen for 5000 ms (inter-probe interval). After the inference probe, the sequences proceeded.

The task included short breaks (30 s) after every 12th presented sequence and a longer break (60 s) between prior and transfer learning. During these breaks, participants received feedback about

their average performance in the experience probe questions, up until this trial. As well as assessing whether participants entertained a factorized representation of the graph factors generating the sequence of compound observations, our transfer learning task allowed us to test a prediction that participants form abstractions of the decomposed or factorized state space, and reuse components of the abstracted generative processes (graph factors) encountered in prior learning in an entirely new context, the transfer learning phase. Importantly, the design also implies a second type of transfer learning, other than reusing a graph factor during the second learning phase: Participants could repurpose their knowledge about the experienced transitions (as assessed by experience probes) to infer never experienced transitions on the graph factors (as assessed by inference probes).
We reasoned that if subjects factorized and abstracted subprocess dynamics underlying their experience, they would then reuse a subprocess that was consistent across prior and transfer learning. During transfer learning, we expected an advantage in predicting 4-cycle graph factor transitions in the 4-cycle prior condition (vs 6-cycle prior condition), and vice versa, enhanced performance in 6-cycle graph factor transitions in the 6-cycle prior condition (vs 4-cycle prior condition) – an interaction effect between condition and probed size.

Behavioral analyses
Data were analyzed in MATLAB 2023b (The MathWorks, Inc., Natick, MA, USA) and RStudio) (1) (version 4.1.0, RStudio Team, Boston, MA) using custom analysis scripts. We defined Bayesian multilevel generalized linear models (GLM), representing different processes that might have generated the observed choice data using the R package rethinking (2, 3). We specified binomial likelihood functions to model the (aggregated) number of correct responses distributed as the proportion of correct probes (binomial distribution parameter *p*) among the number of probes for which a response was recorded (number of probes). We employed sampling-based Bayesian inference to estimate the posterior distribution of linear model parameters comprising different intercept and slope parameters that linearly combine to the binomial distribution parameter *p*. In the below models, μ denotes average/group level effects ("fixed effects"), α and γ denote individual or individually covarying effects ("random effects"). We coded the probed size effect as –.5 (for 4-cycle probes) and as .5 (for 6-cycle probes). A negative value of the parameter estimate indicates higher accuracy for 4-cycle probes. The probe type effect was coded similarly (experience probes as –.5; inference probes as .5). A negative value of the parameter estimate indicates higher accuracy for experience probes. The condition effect was coded as –.5 (for 4-cycle prior condition) and as .5 (for 6-cycle prior condition). A negative value of the parameter estimate indicates higher accuracy for the 4-cycle prior condition.

For each of the below models, we specified weakly informative prior and hyperprior probability distributions (as indicated in the model specifications). Models were passed to RStan (4) using the function "ulam" (rethinking package). We drew 4 x 4000 samples from posterior probability distributions (4 x 1000 warmup samples), using No-U-Turn samplers (NUTS; a variant of Hamiltonian Monte Carlo) in RStan and four independent Markov chains. Quality and reliability of the sampling process were evaluated with the Gelman-Rubin convergence diagnostic measure ($\hat{R} \approx 1.00$) and by visually inspecting Markov chain convergence using trace- and rank-plots. For all models fitted we found $\hat{R} = 1.00$ for all parameters sampled from the posterior distribution. There were no divergent transitions between Markov chains for any of the models reported.

For model comparisons and to find evidence for the best-fitting model for the observed behavioral data, we used Pareto-Smoothed Importance Sampling (PSIS) and the Widely Applicable Information Criterion (WAIC). Parameter estimates were considered non-zero if the Bayesian credible interval (CI) around the parameter did not contain zero.

For hypothesis testing, e.g., in the overall performance analysis, we compared the inverse logit transformed probe type specific parameter estimates in the respective task phases to chance level (probability correct = .50). To this end, we calculated 89%-highest posterior density intervals (89%-HPDI) for each probe type/task phase specific probability correct. The HPDI represents the parameter values most consistent with the data. The HPDI was then compared to a region of

practical equivalence (ROPE), i.e., an interval of parameter values (probability correct = [.45; .55]) representing the null hypothesis of performance being equivalent to chance level/guessing. Specifically, we expected that the 89%-HPDI would not overlap with the ROPE for any of the probe type specific parameter estimates in the respective task phases, allowing us to reject the null hypothesis.

Models for overall performance

To analyze overall performance and to assess evidence that participants could infer and predict never experienced transitions between novel compounds (inference probes), we tested whether overall experience probe and overall inference probe performance during both prior learning and transfer learning phase – regardless of the probed size or condition – was above chance level. To this end and after establishing superior model fits of multilevel models over a single-level model (GLM1, Eq. 1), we fit a multilevel GLM containing individually varying intercepts, covarying probe type (experience probe or inference probe) and task phase specific slope parameters (prior learning phase or transfer learning phase, 4 levels, GLM2, Eq. 2). This model allows variation across subjects, probe types and task phases, and includes the covariation between intercepts and slopes, capturing individual variation in these effects.

GLM1. Overall performance single-level model: One parameter for each probe type in both task phases (*TP/PT*)

$$\begin{gathered} Correct_l \sim Binomial(number\,of\,probes_l,\, p_l) \\ logit(p_l) = \mu_{TP/PT[l]} \\ \mu_{TP/PT[l]} \sim Normal(0,\,1) \end{gathered} \quad \textit{(Eq. 1)}$$

where *Correct* denotes the number of correct probes, distributed as the proportion of correct probes (binomial distribution parameter *p*) among the number of probes for which a response was recorded (number of probes). $TP/PT[l]$ denotes the parameter estimate for the l-th task phase and probe type combination (4 levels).

GLM2. Overall performance multilevel model: Individually varying intercepts, one parameter for each task phase and each probe type, covarying intercept and task phase/probe type effect

$$\begin{gathered} Correct_{l,n} \sim Binomial(number\,of\,probes_{l,n},\, p_{l,n}) \\ logit(p_{l,n}) = \mu_{TP/PT[l]} + \alpha_{subject[n],TP/PT[l]} \\ \begin{bmatrix} \alpha_{n,1} \\ \alpha_{n,2} \\ \alpha_{n,3} \\ \alpha_{n,4} \end{bmatrix} \sim MVNormal\left(\begin{bmatrix} 0 \\ 0 \\ 0 \\ 0 \end{bmatrix},\, S \right) \\ S = \begin{pmatrix} \sigma_{subject} & 0 \\ 0 & \sigma_{TP/PT} \end{pmatrix} R_{TP/PT} \begin{pmatrix} \sigma_{subject} & 0 \\ 0 & \sigma_{TP/PT} \end{pmatrix} \\ \mu_{TP/PT[l]} \sim Normal(0,\,1) \\ \sigma_{subject} \sim Half-Normal(0,\,1) \\ \sigma_{TP/PT} \sim Half-Normal(0,\,1) \\ R_{TP/PT} \sim LKJcorr(2) \end{gathered} \quad \textit{(Eq. 2)}$$

where *MVNormal* is a multivariate normal/Gaussian distribution, the correlation matrix $R_{TP/PT}$ is distributed *as LKJcorr* distribution (Lewandowski-Kurowicka-Joe distribution). We used non-centered parameters for the covariance matrix S (Cholesky decomposition) to facilitate estimation

using sampling-based inference. σ are the variance parameters for the subject specific intercept parameter and task phase/probe type parameter.

Model comparison showed that GLM2 fit the data better than GLM1: Pareto-Smoothed Importance Sampling (PSIS) values ($\pm$ standard error): GLM2 = 11068.1 ($\pm$137.82), GLM1 = 12678.1 ($\pm$170.29); Widely Applicable Information Criterion (WAIC) values ($\pm$ standard error): GLM2 = 10897.6 ($\pm$132.56), GLM1 = 12678.1 ($\pm$170.24).

Models for transfer learning performance

We defined a model space of four Bayesian multilevel (statistical models, GLM3 - GLM6, Eq. 3-6) generalized linear models (cf. (5)). Both experience and inference probe correct responses were analyzed in a joint model to reduce the number of tests of the same statistical hypothesis and to increase statistical power (a joint model should capture within-subject performance ability and correlations across both probe types more adequately and explicitly than two separate models for both probe types). To test our main hypothesis, that participants factorized and abstracted structure underlying their experience during prior learning and reuse an abstracted graph factor consistent across prior and transfer learning, we focused on transfer learning phase behavior. We expected an advantage in predicting 4-cycle graph factor transitions in the 4-cycle prior condition (vs 6-cycle prior condition), and vice versa, enhanced performance in 6-cycle graph factor transitions in the 6-cycle prior condition (vs 4-cycle prior condition). Formally, this pattern of responses should reflect in an interaction effect between condition and probe question probed size. We therefore predicted that the best-fitting model for the observed behavioral data (as found in model comparison) would feature a non-zero interaction effect between condition and probed size, suggesting that this effect captures important unique variance in explaining participants' choice behavior.

GLM3. Individually varying intercepts model. This model assumes that correct responses are invariant across probed sizes, probe types, and conditions.

$$
\begin{aligned}
Correct_n &\sim Binomial(number\,of probes_n,\ p_n) \\
logit(p_n) &= \mu_{\alpha} + z_{subject[n]} * \alpha_{sigma} \\
\mu_{\alpha} &\sim Normal(0, 1) \\
\alpha_{sigma} &\sim Half-Normal(0, 1) \\
z_{subject[n]} &\sim Normal(0, 1)
\end{aligned}
\qquad \textit{(Eq. 3)}
$$

where *Correct* denotes the number of correct probes, distributed as the proportion of correct probes (binomial distribution parameter *p*) among the number of probes for which a response was recorded (number of probes), *n* denotes the parameter estimate for the n-th subject. Note that the model is reparametrized to allow sampling from a standard normal posterior distribution. α_{sigma} denotes the standard deviation of the reparameterized normal distribution of the varying intercepts parameter α.

GLM4. Individually varying intercepts, main effect of probe type (*PT*), covarying intercept and probe type effect

$$Correct_{j,n} \sim Binomial\left(number\,of\,probes_{j,n},\ p_{j,n}\right)$$
$$logit(p_{j,n}) = \mu_{\alpha} + \mu_{PT[j]} + \alpha_{subject[n],PT[j]}$$
$$\begin{bmatrix} \alpha_{n,1} \\ \alpha_{n,2} \end{bmatrix} \sim MVNormal\left(\begin{bmatrix} 0 \\ 0 \end{bmatrix},\ S \right)$$
$$S = \begin{pmatrix} \sigma_{subject} & 0 \\ 0 & \sigma_{PT} \end{pmatrix} R_{PT} \begin{pmatrix} \sigma_{subject} & 0 \\ 0 & \sigma_{PT} \end{pmatrix} \qquad \textit{(Eq. 4)}$$
$$\mu_{\alpha} \sim Normal(0, 1)$$
$$\mu_{PT} \sim Normal(0, 1)$$
$$\sigma_{subject} \sim Half-Normal(0, 1)$$
$$\sigma_{PT} \sim Half-Normal(0, 1)$$
$$R \sim LKJcorr(2)$$

where *MVNormal* is a multivariate normal/Gaussian distribution, the correlation matrix R_{PT} is distributed *as LKJcorr* distribution (Lewandowski-Kurowicka-Joe distribution). We used non-centered parameters for the covariance matrix S (Cholesky decomposition) to facilitate estimation using sampling-based inference. j denotes the parameter estimate for the j-th probe type (experience or inference probe). $\sigma$are the variance parameters for the subject specific intercept parameter and probe type parameter.

GLM5. Individually varying intercepts, main effects of probed size, probe type and condition, interaction effect of probed size x condition (*SIZE x COND*), covarying intercept and probed size, probe type effect

$$Correct_{i,j,k,n} \sim Binomial\left(number\,of\,probes_{i,j,k,n},\ p_{i,j,k,n}\right)$$

$$logit\left(p_{i,j,k,n}\right) = \mu_{\alpha} + \mu_{SIZE[i]} + \mu_{PT[j]} + \mu_{COND[k]} + \alpha_{subject[n],SIZE[i]} + \gamma_{subject[n],PT[j]} + \mu_{SIZEx\,COND}$$

$$\begin{bmatrix} \alpha_{n,1} \\ \alpha_{n,2} \end{bmatrix} \sim MVNormal\left(\begin{bmatrix} 0 \\ 0 \end{bmatrix}, S_{SIZE}\right)$$

$$\begin{bmatrix} \gamma_{n,1} \\ \gamma_{n,2} \end{bmatrix} \sim MVNormal\left(\begin{bmatrix} 0 \\ 0 \end{bmatrix}, S_{PT}\right)$$

$$S_{SIZE} = \begin{pmatrix} \sigma_{subject} & 0 \\ 0 & \sigma_{SIZE} \end{pmatrix} R_{SIZE} \begin{pmatrix} \sigma_{subject} & 0 \\ 0 & \sigma_{SIZE} \end{pmatrix}$$

$$S_{PT} = \begin{pmatrix} \sigma_{subject} & 0 \\ 0 & \sigma_{PT} \end{pmatrix} R_{PT} \begin{pmatrix} \sigma_{subject} & 0 \\ 0 & \sigma_{PT} \end{pmatrix} \qquad \text{(Eq. 5)}$$

$$\mu_{\alpha} \sim Normal(0, 1)$$
$$\mu_{SIZE[i]} \sim Normal(0, 1)$$
$$\mu_{PT[j]} \sim Normal(0, 1)$$
$$\mu_{COND[k]} \sim Normal(0, 1)$$
$$\mu_{SIZEx\,COND} \sim Normal(0, 1)$$
$$\sigma_{subject} \sim Half-Normal(0, 1)$$
$$\sigma_{SIZE} \sim Half-Normal(0, 1)$$
$$\sigma_{PT} \sim Half-Normal(0, 1)$$
$$R_{SIZE} \sim LKJcorr(2)$$
$$R_{PT} \sim LKJcorr(2)$$

where *k* denotes the parameter estimate for the k-th condition (4-cycle prior condition or 6-cycle prior condition), i denotes the parameter estimate for the i-th probed size (4-cycle probe or 6-cycle probe)

GLM6. Individually varying intercepts, main effects of probed size, probe type and condition, interaction effect of probed size x condition and probed size x probe type x condition (*SIZE x PT x COND*), covarying intercept and probed size, probe type effect

$$Correct_{i,j,k,n} \sim Binomial\left(number\ of probes_{i,j,k,n},\ p_{i,j,k,n}\right)$$

$$logit\left(p_{i,j,k,n}\right) = \mu_{\alpha} + \mu_{SIZE[i]} + \mu_{PT[j]} + \mu_{COND[k]} + \alpha_{subject[n],SIZE[i]} + \gamma_{subject[n],PT[j]} + \mu_{SIZEx\,COND} + \mu_{SIZExPTxCOND}$$

$$\begin{bmatrix}\alpha_{n,1}\\ \alpha_{n,2}\end{bmatrix} \sim MVNormal\left(\begin{bmatrix}0\\0\end{bmatrix}, S_{SIZE}\right)$$

$$\begin{bmatrix}\gamma_{n,1}\\ \gamma_{n,2}\end{bmatrix} \sim MVNormal\left(\begin{bmatrix}0\\0\end{bmatrix}, S_{PT}\right)$$

$$S_{SIZE} = \begin{pmatrix}\sigma_{subject} & 0\\ 0 & \sigma_{SIZE}\end{pmatrix} R_{SIZE} \begin{pmatrix}\sigma_{subject} & 0\\ 0 & \sigma_{SIZE}\end{pmatrix}$$

$$S_{PT} = \begin{pmatrix}\sigma_{subject} & 0\\ 0 & \sigma_{PT}\end{pmatrix} R_{PT} \begin{pmatrix}\sigma_{subject} & 0\\ 0 & \sigma_{PT}\end{pmatrix} \qquad \textit{(Eq. 6)}$$

$$\mu_{\alpha} \sim Normal(0, 1)$$
$$\mu_{SIZE[i]} \sim Normal(0, 1)$$
$$\mu_{PT[j]} \sim Normal(0, 1)$$
$$\mu_{COND[k]} \sim Normal(0, 1)$$
$$\mu_{SIZEx\,COND} \sim Normal(0, 1)$$
$$\mu_{SIZExPTxCOND} \sim Normal(0, 1)$$
$$\sigma_{subject} \sim Half-Normal(0, 1)$$
$$\sigma_{SIZE} \sim Half-Normal(0, 1)$$
$$\sigma_{PT} \sim Half-Normal(0, 1)$$
$$R_{SIZE} \sim LKJcorr(2)$$
$$R_{PT} \sim LKJcorr(2)$$

For the analysis on all probes during transfer learning, a multilevel GLM featuring individually varying intercepts, main effects of probed size and probe type, as well as covariation with the individually estimated intercept parameters, a main effect of condition, and an interaction effect of probed size x condition (GLM5) and a multilevel GLM featuring all of the above effects and a three-way interaction effect of probed size x probe type x condition (GLM6) showed almost identical model fits (PSIS values (± standard error): GLM5 = 4460.8 (±55.18), GLM6 = 4463.2 (±56.22); WAIC values (± standard error): GLM6 = 4244.4 (±48.74), GLM5 = 4245.6 (±47.80)). All other GLMs explained the data less well than GLM5 or GLM6. Crucially, there was strong model evidence that GLMs featuring varying intercepts alone (e.g., GLM3), representing the alternative hypothesis of participants entertaining a compound representation of their experience, was much worse explaining observed behavioral data. Neither of the two best-fitting models (GLM5 nor GLM6) captured the behavioral data better than the respective other across both model comparison metrics. There was no consistent model evidence favoring the more complex model (GLM6), suggesting that adding a higher-order interaction effect, describing the modulation of the observed probed size x condition interaction through the probe type did not improve model fits. We thus considered the less-parameterized GLM5 to provide the more parsimonious explanation for the data – i.e., that the observed interaction effect of probed size x condition was present and not meaningfully different in experience and inference probes. As in the analysis

across all probes, GLM5 also provided the most parsimonious explanation for the observed data (PSIS values (± standard error): 1155.7 (18.82); WAIC values (± standard error): 1153.1 (18.75)). Crucially, there was strong model evidence that GLMs only featuring varying intercepts (e.g., GLM3), representing the alternative hypothesis of participants entertaining a compound representation of their experience, were much inferior at explaining observed behavioral data (PSIS (± standard error): 1207.9 (12.20), WAIC (± standard error): 1207.9 (12.19)).

Computational modeling

Our model space included three successor representation models described below. For each model, different successor representation matrices were learned per session (prior and transfer learning). This reflects the design of the behavioral task – entirely different and unrelated compound images and features were employed in the prior and transfer learning phases.

The first model was a successor compound model (**M1**, Eq. 7), learning an expected future compound occupancy from the sequence of compound images, and storing this quantity in the successor compound matrix (M). Following a transition from compound s_t to s_{t+1} the following update is executed (for any compound s'):

$$M_{t+1}(s_t, s') = M_t(s_t, s') + \alpha\left[I(s_t = s') + \gamma M_t(s_{t+1}, s') - M_t(s_t, s')\right] \quad \textit{(Eq. 7)}$$

where α is a learning rate parameter controlling the rate of update of expected future compound occupancy; γ is a discounting parameter (fixed to 0.9 in all simulations and during model fitting); I is 1 if the equality inside the () holds and otherwise 0.

Choice is this model governed by a Softmax function. Specifically, presented with a compound probe P and two possible "next step" compounds A and B the probability to choose compound A was:

$$P(A) = \frac{1}{1 + exp\left((M(P,B) - M(P,A)) / \tau\right)} \quad \textit{(Eq. 8)}$$

Where τ is the temperature parameter, determining the degree of stochasticity in choices, and M is the successor representation matrix at the time a probe was queried.

Our second model, the successor feature model (**M2**), learned expectations about the sequences of features composing the compound image sequence. For each of the two currently presented features, the model learned an association with upcoming features. Note this model is not endowed with knowledge that two features evolve according to separate dynamical components. Thus, learning in this model is achieved by calculating a state prediction error, following a transition between compounds, and distributing it equally between the two current features. Specifically, following the observation of a transition between a feature pair $\{s_{t,1}, s_{t,2}\}$ to a pair $\{s_{t+1,1}, s_{t+1,2}\}$ the following update was executed for each feature s' and i = 1,2:

$$M_{t+1}(s_{t,i}, s') = M_t(s_{t,i}, s') + \frac{\alpha}{2}\left[I(s_{t,1} = s') + I(s_{t,2} = s') + \gamma(M(s_{t+1,1}, s') + M(s_{t+1,2}, s')) - (M(s_{t,1}, s') + M(s_{t,2}, s'))\right]$$

(Eq. 9)

Choice in this model is governed by a Softmax function. Specifically, presented with a compound probe $P = \{P_1, P_2\}$ and two possible "next step" compounds $A = \{A_1, A_2\}$ and $B = \{B_1, B_2\}$ the probability to choose A was:

$$P(A) = \frac{1}{1 + exp\left((M(P_1,B_1) + M(P_1,B_2) + M(P_2,B_1) + M(P_2,B_2) - (M(P_1,A_1) + M(P_1,A_2) + M(P_2,A_1) + M(P_2,A_2))) / \tau\right)}$$

(Eq. 10)

The third successor feature model (**M3**) was an extension of the successor feature model (**M2**), but importantly, incorporated transfer learning abilities. **M3** integrated information learned during the prior learning phase with information acquired during transfer learning, by combining knowledge represented in the successor feature matrices learned during each session (M_{Prior} , $M_{Transfer}$). Importantly, these two successor feature matrices were learned in the very same way as in M2. However, when a probe was presented, the choice was not solely governed by $M_{Transfer}$, but instead, the model could reuse the previously learned structure M_{Prior}. This is possible because both matrices consist of sub-matrices that could be aligned productively (i.e., 4-

cycle to 4-cycle or 6-cycle to 6-cycle). To do so, one needs to find a 1:1 mapping from one feature space to the other, which amounts to reordering the rows and columns of the prior matrix in order to best fit to the current matrix $M_{Transfer}$. An optimal mapping, f^* can be found by minimizing the distance in Equation 11 between the "remapped" (end of session) prior learning SR and the current transfer learning SR:

$$f^* = \text{argmin}_f \sum s, s' \, [M_{transfer}(s, s') - M_{prior}(f(s), f(s))]^2 \qquad \textit{(Eq. 11)}$$

Specifically, the algorithm searches the space of permutations to find an optimal mapping via a simulated annealing procedure. We initialize a random permutation f and iterate two steps: 1) Propose a new permutation $f_{proposal}$ by swapping to elements of the current f. 2) Accept the proposal with probability $p_{accept} = exp(\,(d(f) - d(f_{proposal}))\,/\,T\,)$, where d is the distance function in Equation 11 and T is a temperature parameter which follows a decreasing schedule (negative exponential) over the course of the optimization, injecting some degree of randomness into the acceptance (to prevent the algorithm from getting stuck in a local minimum). At the end of this procedure f represents the mapping that minimizes the distance and thus best aligned the two SR matrices. Based on the mapping f a choice was driven by "mixing" information from the current $M_{transfer}$ and the "remapped'" (end of session) M_{prior}. Specifically, we define the "Mixed SR Matrix" (denoted M) by:

$$M(s, s') = (1 - \omega) \cdot M_{transfer}(s, s') + \omega \cdot M_{prior}(f(s), f(s)) \qquad \textit{(Eq. 12)}$$

where, ω is a weighting parameter, quantifying the strength of prior knowledge reuse. The next state probe was chosen according to Eq. 10 but with M – the mixed SR Matrix.

We formulated 4 different versions of each model (**M1-3**) by considering all possibilities of combining the number of learning rates (one or two) and temperature parameters (one or two). Versions with two learning rates captured a phase (prior vs. transfer learning) influence whereas versions with two temperatures expressed a probe-type (experience vs. inference) influence. Across all prior simulations, we used the following parameter values:

$$\alpha_{prior} = 0.01;\ \alpha_{transfer} = 0.05; \tau_{experience} = 0.3; \tau_{inference} = 0.6; \omega = 0.25; \gamma = 0.9$$

Parameter optimization, model comparison and posterior simulations

To find the free parameters that best described participants' behavior, for **M1-3** we minimized the negative log likelihood estimate (−LLE) of the parameters given the participant's choices, jointly across both prior and transfer learning (Eq. 13).

$$-LLE = -\Sigma log(P_{choice,\,probe}) \qquad \textit{(Eq. 13)}$$

Where $P_{choice,\,probe}$ is the probability of a given model making the observed participant's choice on a given probe. Parameters were optimized using constrained non-linear optimization using the MATLAB function fmincon. To decrease the probability of obtaining a local minimum in the parameter space geometry, we repeated the parameter optimization process 20 times for each subject using randomly initialized starting values for the optimizer (for **M1** and **M2**). Since model **M3** was a nested version of **M2** (setting $\omega = 0$ reduces **M3** to **M2** – no reuse of prior knowledge occurs), we used the optimized parameter estimates (α and τ) of the corresponding variant of **M2** as starting values for fitting parameters in **M3** (only randomly initializing ω).

For model comparison, we used the Widely Applicable Information Criterion (WAIC).

We used the individually best-fitting parameter estimates for each model to run posterior simulations. To this end, the obtained parameter estimates for each participant were used to run 1000 forward model simulations to generate choices for the respectively presented probe questions. Correctness of these simulated choices was scored against the ground truth correct answers (similar as with the observed choices). We used the average probability of giving a correct answer for each respective probed size during transfer learning to recover the crucial feature observed in the behavioral data: The interaction effect between probed size and condition. To visualize this effect, we computed the mean difference between 4-cycle and 6-cycle probe performance between conditions.

Supporting Information Text

Prior knowledge supports rapid relearning of subprocesses

We investigated how transfer learning effects emerge across time. Under the assumption that participants reuse abstracted subprocesses compositionally, the benefits in re-encountering a previously experienced graph component should, in principle, already manifest in the first trials of the transfer learning phase. This is because in a new scenario reusing subprocess dynamics increase the chances of discovering the same subprocesses encountered during prior learning. To test this, we repeated the same analysis as above, but now restricted to the first two transfer learning probe questions. Note, these featured one probe question testing knowledge of the 4-state cycle and one probe question testing knowledge of the 6-state cycle. We found that even in the first two trials, participants performed better on probe questions about the graph factor they had experienced in prior learning (Supplementary Fig. 3A). Consistent with the analysis across all trials in transfer learning, the most parsimonious model again (see Supplementary Materials and Methods for model comparison) featured a non-zero interaction effect between probed size and condition (interaction effect: $\mu_{SIZExCOND}$ = .48, CI = [.21; .76], Supplementary Fig. 3B, bottom right panel). In post-hoc contrasts disentangling this effect, we found that the interaction was driven by a higher average probability of giving correct answers in 4-state cycle probes in the 4-state cycle prior condition (vs 6-cycle prior condition, mean difference of 10,000 posterior samples: .37, CI = [.09; .67]). However, we only detected a trend towards better performance in 6-state cycle probes in the 6-state cycle prior condition (vs 4-cycle prior condition, mean difference of 10,000 posterior samples: .09, CI = [-.05; .33]). This analysis is consistent with prior learning abstracted dynamics being reused to support rapid new learning, even with limited exposure to novel environments. The temporal dynamics of compositional generalization of abstracted subprocesses support a model that assumes reuse of subprocess dynamics encountered during prior learning.

Computational modeling: Apriori simulations

We leveraged apriori simulations using three classes of agents performing the experiment to further detail cognitive strategies that could be used to solve the task (Supplementary Fig. 5). To this end, we simulated 250 model instantiations per computational model with fixed parameters (see Methods for details), ensuring matching parameter values across prior learning conditions.

Models were presented with identical sequences of compounds presented to human participants and made choices in relation to both experience and inference probes.

While all successor predictive models without transfer learning abilities (**M1-M2**) performed well on experience probes (Supplementary Fig. 5D/E), only **M2** and **M3** showed above-chance performance for inferring transitions between unobserved compositions (inference probes). Crucially, none of the models **M1-2** predicted enhanced transfer learning performance for the graph factor that was consistent across prior and transfer learning (Supplementary Fig. 5D/E).

Only **M3** showed specifically increased transfer learning performance for the graph factor that was consistent across prior and transfer learning in both conditions (Supplementary Fig. 5F). These apriori simulation results suggest that a successor feature model endowed with transfer

learning and knowledge reuse abilities was capable of representing subprocess dynamics underlying the sequence of compound images and could reuse this prior knowledge compositionally during transfer learning.
These apriori simulations suggest that the three candidate models, incorporating different assumptions about the underlying principles of the learning process would make highly distinct behavioral predictions, and could be differentiated.

In both samples, we observed that a group difference on 6-cycle inference probes was unexpectedly of negligible magnitude and did not align with the robust effect observed for experience probes. A potential explanation for the absence of a difference could stem from capacity limitations on learning and storing relational sequences in memory. This is akin to known capacity limits on the number of stimulus-outcome associations that can be held in working memory as observed in model-free reinforcement learning tasks, putatively related to genetic phenotypes of basal ganglia functioning (6) and fronto-parietal neural activation sensitive to the stimulus set-size (7). However, this account does not explain the specific behavioral differences observed across probe types in our study, since under a working memory capacity limitation, we would expect similar effect size differences for inference as for experience probes. We speculate that experience and inference probes may engage dissociable cognitive processes – the former relying on predictive representations acquired from experience (and hence a mixture of compound and feature representations) with the latter relying on participants' ability to perform a mental manipulation on the learned representation of independent feature transitions (necessitating representing feature transitions). We formally tested this hypothesis and found that participants reporting explicit knowledge of generative subprocesses tin post-experimental questionnaires (*N* = 172) showed higher performance in both experience and inference probe questions than those lacking explicit knowledge (Supplementary Fig. 4). In the explicit knowledge group, an interaction effect was observed for inference probes and for experience probes (Supplementary Fig. 4), suggesting that inferential reasoning using the learned representations may depend on explicit knowledge of underlying subprocesses.

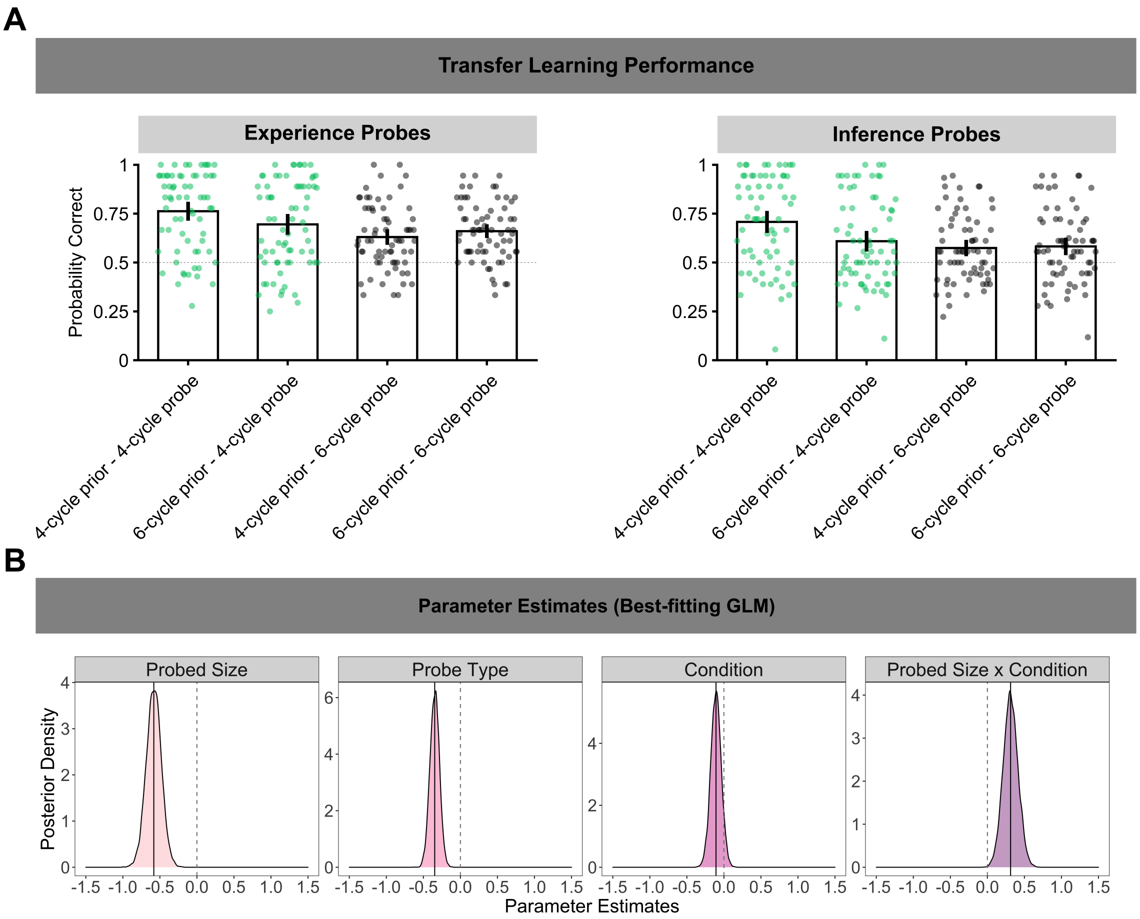

Supplementary Figure 1. Transfer learning performance in an independent sample.

A) Aggregated probabilities of correct answers during the transfer learning phase, separately for both experience (left) and inference probes (right) as a function of probed size (4-state cycle (green) vs 6-state cycle (black)) and condition (4-state cycle prior vs 6-state cycle prior) in an independent sample of healthy volunteers (*N* = 137). Each dot represents one participant, bars represent the arithmetic mean of the distribution, error bars depict the 95% confidence interval around the mean and the dashed gray line represents chance level point estimate (probability correct = 0.5).

B) Posterior density plots for each parameter estimate (posterior mean = black line) for the best-fitting GLM. The dashed vertical line represents a zero effect. In probed size and probe type effects, parameter estimates below zero indicate higher accuracy in the 4-cycle probes (vs. 6-cycle probes) and in experience probes (vs. inference probes), respectively. In the condition effect, parameter estimates below zero indicate higher accuracy in the 4-cycle prior condition (vs. 6-cycle prior condition).

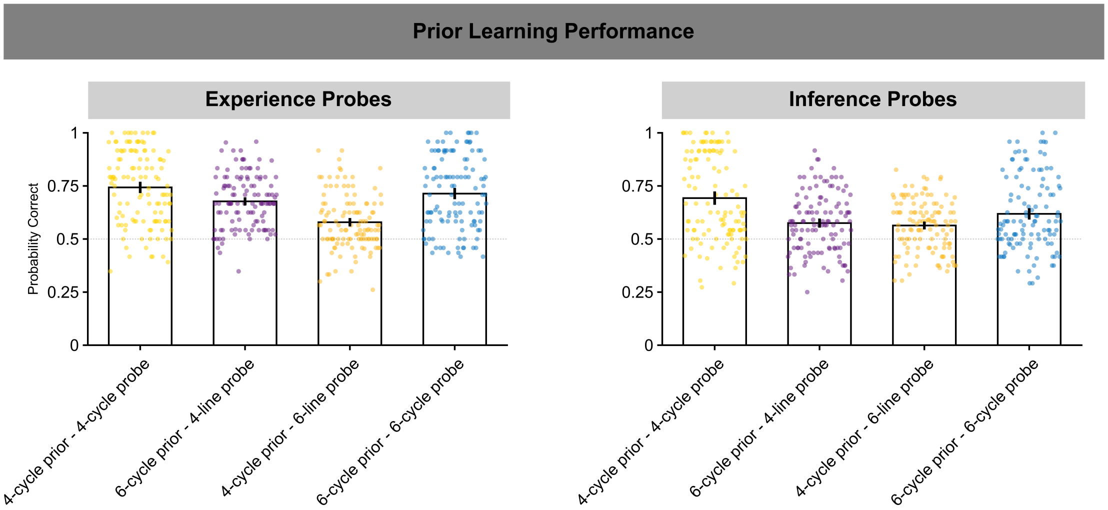

Supplementary Figure 2. Prior learning performance.
Aggregated probabilities of correct answers during the prior learning phase, presented separately for both experience (left) and inference probes (right) as a function of probed size and condition (4-state cycle prior condition: 4-state cycle (yellow) and 6-state path-graph (gold) vs 6-state cycle prior condition: 4-state path-graph (purple) 6-state cycle (blue)), in the main sample of healthy volunteers (N = 238) Each dot represents one participant, bars represent the arithmetic mean of the distribution, error bars depict the 95% confidence interval around the mean and the dashed gray line represents chance level point estimate (probability correct = 0.5).

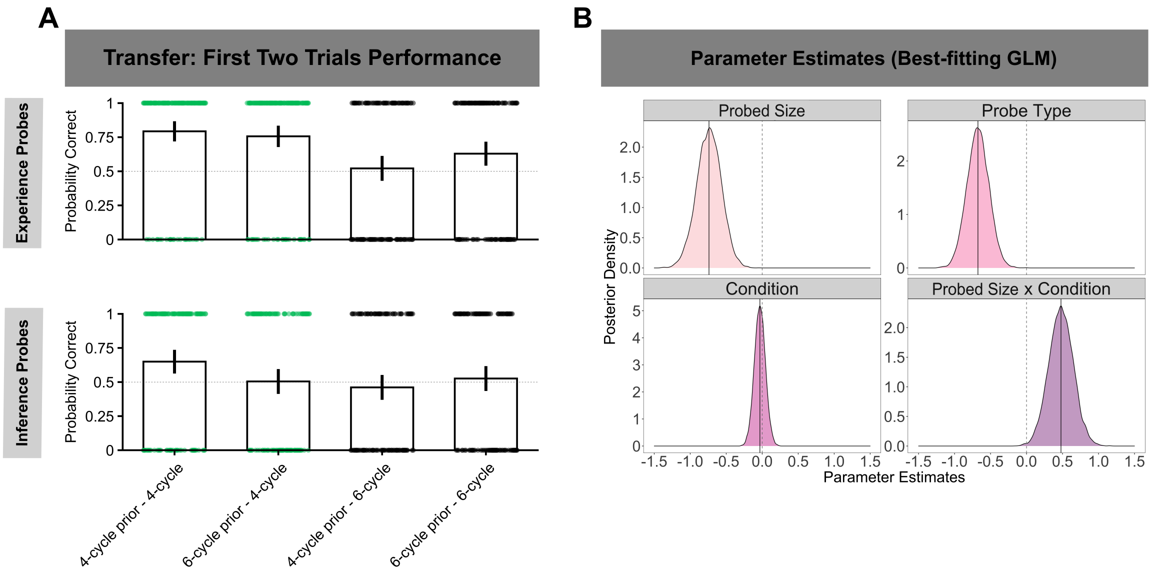

Supplementary Figure 3. Prior knowledge supports rapid structural inference during initial trials of the transfer learning phase.

A) Probabilities of correct answers during the first two trials in the transfer learning phase, separately for experience (top) and inference probes (bottom). Depicted is the probability of correct answers, broken up as a function of probed size (4-state cycle (green) vs 6-state cycle (black)) and condition (4-state cycle prior vs 6-state cycle prior). Each dot represents one participant. Note that the transfer learning phase was designed such that the first two trials featured one probe testing knowledge of the 4-state cycle and one probe testing knowledge of the 6-state cycle. Hence, for each participant, per probed size (4-state cycle or 6-state cycle, respectively), the accuracy score can either be 0 or 1. Bars represent group means, error bars depict the 95% confidence interval around the mean and the dashed gray line represents chance level point estimate (probability correct = 0.5).

B) Posterior density plots for each parameter estimate (posterior mean = black line) for the best-fitting GLM. The dashed vertical line represents a zero effect. In probed size and probe type effects, parameter estimates below zero indicate higher accuracy in the 4-cycle probes (vs. 6-cycle probes) and in experience probes (vs. inference probes), respectively. In the condition effect, parameter estimates below zero indicate higher accuracy in the 4-cycle prior condition (vs. 6-cycle prior condition). The positive interaction effect suggests selectively higher accuracy in 4-cycle probes in the 4-cycle prior condition and higher accuracy in 6-cycle probes in the 6-cycle prior condition.

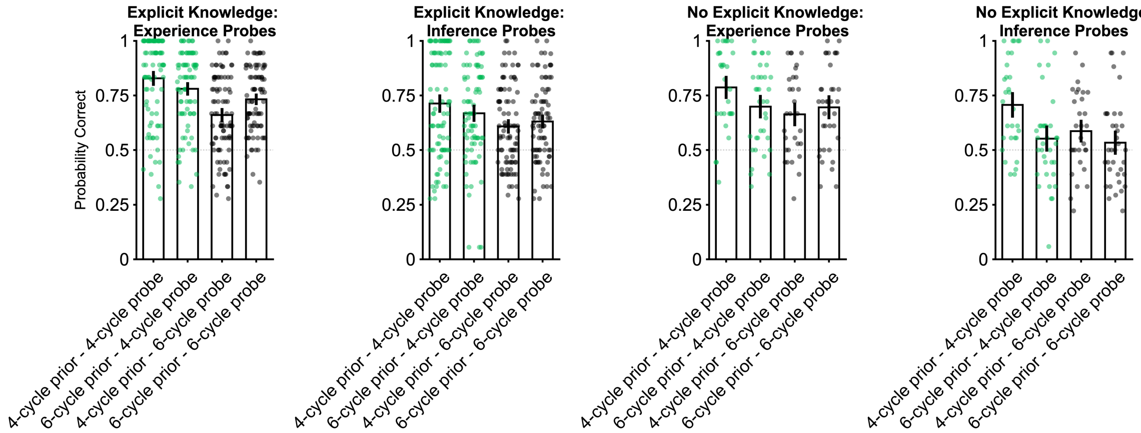

Supplementary Figure 4. Explicit knowledge of subprocesses affects transfer learning performance.

Aggregated probabilities of correct answers during the transfer learning phase, separately for both experience (left) and inference probes (right) as a function of probed size (4-state cycle (green) vs 6-state cycle (black)) and condition (4-state cycle prior vs 6-state cycle prior) in the main sample of healthy volunteers (*N* = 238). Performance is split by reported explicit knowledge of subprocesses generating the observed compound stimulus transitions in post-experimental questionnaires (*N* = 172 participants reported explicit knowledge). Average performance was increased in the explicit knowledge group. Each dot represents one participant, bars represent the arithmetic mean of the distribution, error bars depict the 95% confidence interval around the mean and the dashed gray line represents chance level point estimate (probability correct = 0.5).

Separate GLMs for both groups of participants (explicit vs no explicit knowledge) revealed an interaction effect in both groups (explicit: $\mu_{SIZExCOND}$ = .28, CI = [.21; .35]; no explicit: $\mu_{SIZExCOND}$ = .30, CI = [.15; .44]). However, post-hoc tests revealed that only in the explicit knowledge group average accuracy was higher for 4-cycle probes in the 4-cycle prior condition (vs 6-cycle prior condition; mean difference of 10,000 posterior samples: .24 CI = [.14; .34]) whereas for 6-cycle probes, performance was superior in the 6-state cycle prior condition (vs 4-cycle prior condition; mean difference of 10,000 posterior samples: .19, CI = [.11; .28]). In the no explicit knowledge group only the 4-cycle probes showed higher performance in the 4-cycle prior condition (vs 6-cycle prior condition; mean difference of 10,000 posterior samples: .52 CI = [.38; .66]) whereas for 6-cycle probes, performance was numerically **lower** in the 6-state cycle prior condition (vs 4-cycle prior condition; mean difference of 10,000 posterior samples: -.06, CI = [-.19; .07]), suggesting no evidence for a condition difference on 6-cycle probes in the group without explicit knowledge

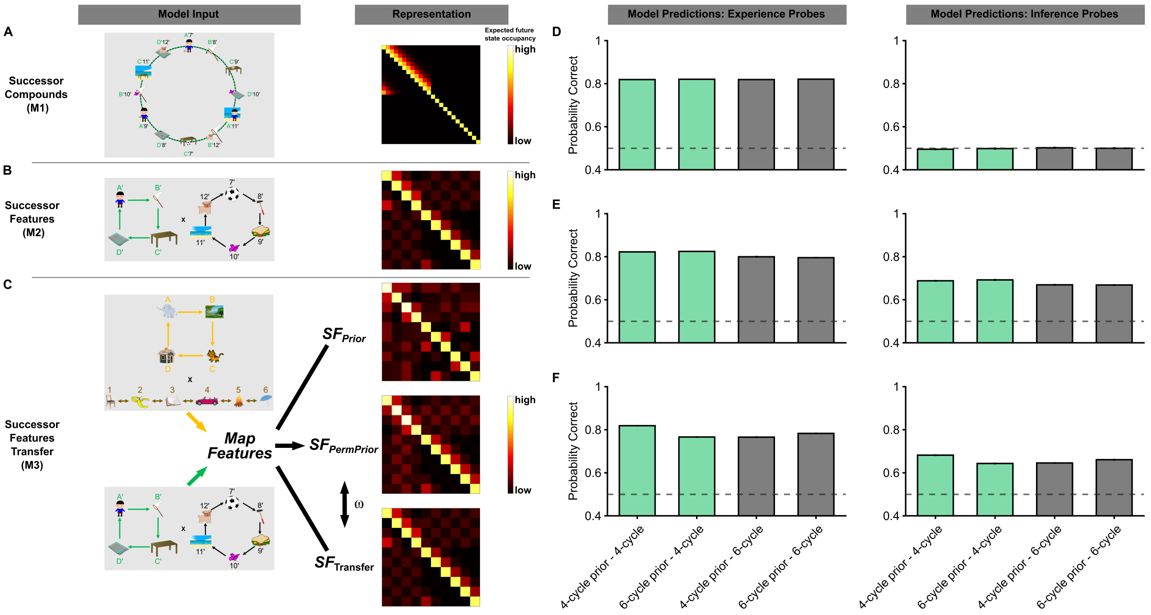

Supplementary Figure 5. Representations and apriori computational model simulations.

A) A successor feature model (**M1**) learned an expected future state occupancy from the sequence of unique compound states and stored this quantity in a successor compound matrix (Representation column). Each row and column represent one compound state. Depicted is the expected, discounted future compound state, occupancy after completion of all trials in transfer learning. Top rows and leftward columns correspond to experience compounds whereas bottom rows/rightward columns correspond to inference compounds. Note that because inference states are never experienced during sequence learning they contribute 0 successor entries.

B) A successor feature model (**M2**) learned expectations about the sequences of features in each graph factor separately (same updating mechanism as **M1**, but operated over two separate features, for the 4-state and 6-state factor separately). Depicted is the expected, discounted future, feature occupancy after completion of all trials in transfer learning. Each row and column represent one feature. The 4 topmost rows and leftmost columns represent features associated with the 4-cycle factor whereas remaining rows/columns represent features of the 6-cycle factor. Note that because this model does not realize that features are arranged into two separate sequences, non-diagonal blocks contribute non-zero successor entries.

C) The successor feature transfer model (**M3**) uses the same updating mechanisms as **M2** but incorporates transfer learning abilities. **M3** leverages a permutation algorithm that searches for the optimal mapping between states/features encountered during prior learning and those observed in transfer learning to map prior knowledge onto new experiences. The permuted prior learning successor feature representation, and newly acquired transfer learning successor feature representation, are weighted by a weighting parameter ω to make experience and inference probe choices.

D) **M1** (2α / 2τ model variant) predictions derived from apriori simulations for Experience Probes (left) and Inference Probes (Right) during the Transfer Learning phase.

E) **M2** (2α / 2τ model variant) predictions.

F) **M3** (2α / 2τ model variant) predictions. In D), E), and F), bars represent the mean, error bars are the 95% confidence of the mean. Per model, we present the probability of giving correct answers, averaged over 20 simulations.

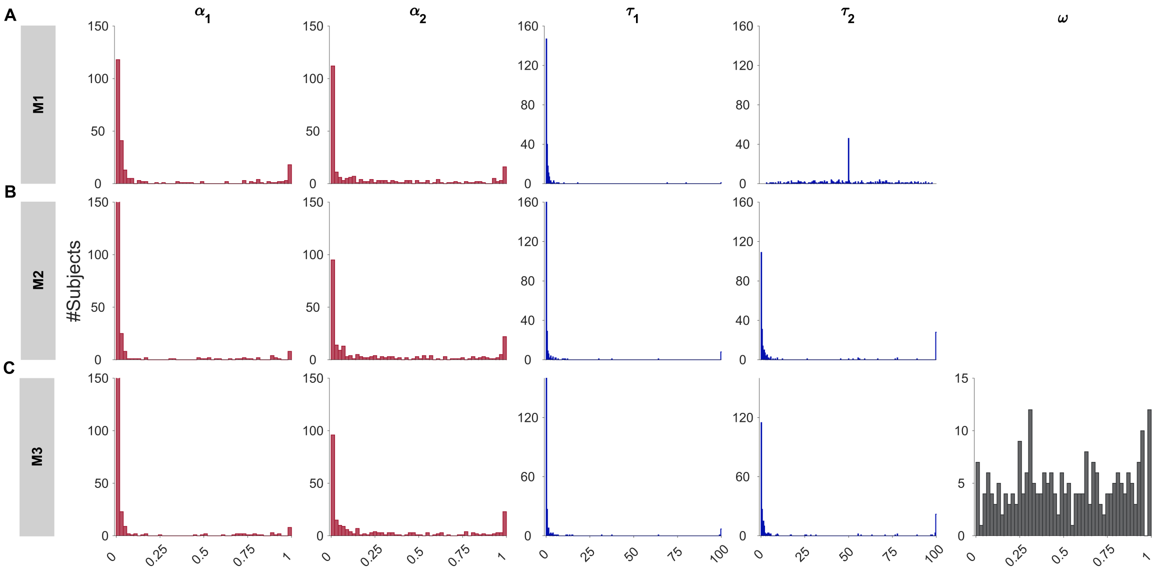

Supplementary Figure 6. Distributions of computational parameter estimates.

Histograms of individually best-fitting parameter estimates (only depicting the best-fitting 2α / 2τ variants of models, for simplicity) obtained by fitting the models to observed behavioral data using maximum likelihood estimation for the successor compound model (**M1,** A), the successor feature model (**M2,** B), and the successor feature transfer model (**M3,** C), across different versions of the models (1α / 2α learning rate parameters, depicted in red, 1τ / 2τ Softmax stochasticity parameters, depicted in blue, and transfer learning parameter ω, depicted in dark gray).

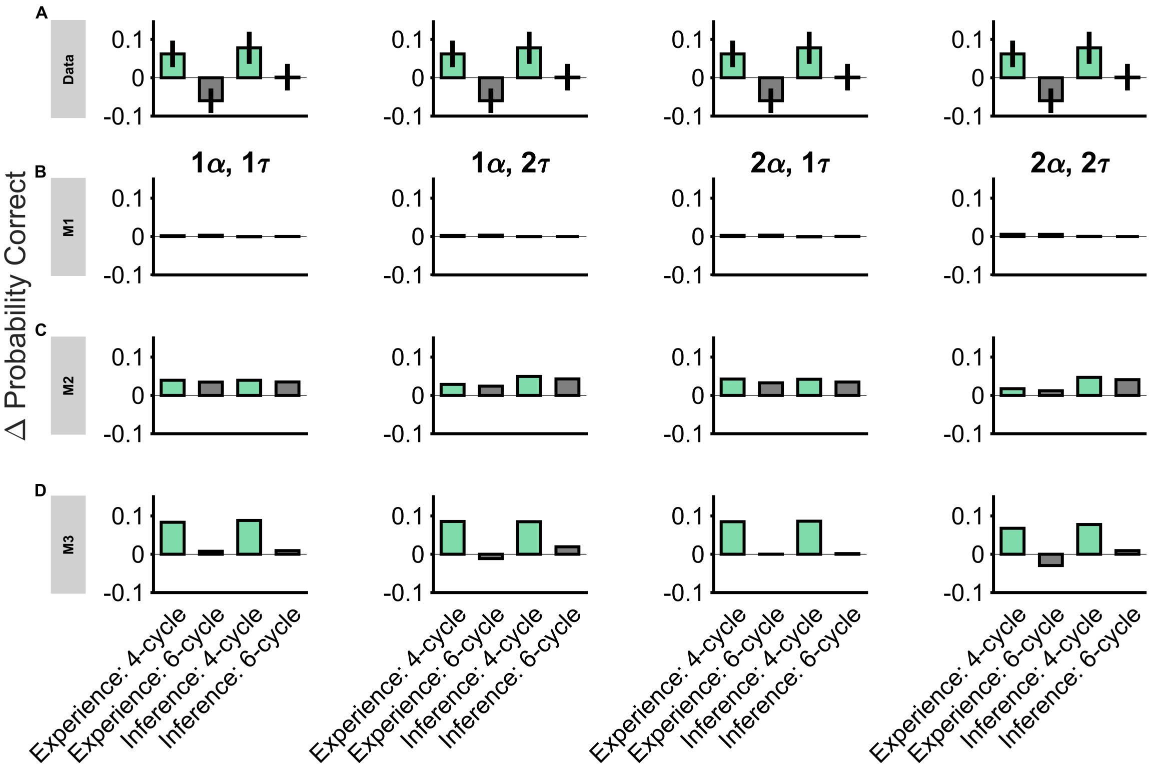

Supplementary Figure 7. Posterior computational model simulations.

Difference between the average probabilities of correct answers in transfer learning between prior learning conditions (4-cycle prior condition minus 6-cycle prior condition) as a function of probed size (4-cycle: green; 6-cycle: black) and probe type (experience vs inference). The observed data (**A**) was compared to simulated choice probability differences (**M1-M3, B-D**) for 4 model variants with different parameterizations per model: 1α / 2α, 1τ / 2τ (represented in different columns). Results were averaged across 1000 posterior simulations. Bar plots represent the mean, error bars are the standard error of the difference of the condition means.

References

1. RStudioTeam, RStudio: Integrated Development for R. [Preprint] (2019). Available at: http://www.rstudio.com/.

2. R. McElreath, rethinking: Statstical rethinking book package. [Preprint] (2020).

3. R. McElreath, *Statistical Rethinking*, 2nd Ed. (CRC Press, 2020).

4. Stan Development Team, RStan: the R interface to Stan. [Preprint] (2020). Available at: http://mc-stan.org/.

5. L. Luettgau, E. Porcu, C. Tempelmann, G. Jocham, Reinstatement of Cortical Outcome Representations during Higher-Order Learning. *Cerebral Cortex* **32**, 93–109 (2022).

6. A. G. E. Collins, M. J. Frank, How much of reinforcement learning is working memory, not reinforcement learning? A behavioral, computational, and neurogenetic analysis. *European Journal of Neuroscience* **35**, 1024–1035 (2012).

7. A. G. E. Collins, B. Ciullo, M. J. Frank, D. Badre, Working memory load strengthens reward prediction errors. *Journal of Neuroscience* **37**, 4332–4342 (2017).
